# TEGAA: transformer-enhanced graph aspect analyzer with semantic contrastive learning for implicit aspect detection

**DOI:** 10.3389/frai.2026.1666674

**Published:** 2026-02-04

**Authors:** Piyush Kumar Soni, Radhakrishna Rambola

**Affiliations:** SVKM’S NMIMS, Mukesh Patel School of Technology Management and Engineering, Shirpur, India

**Keywords:** attention sink, context-aware graph attention, contrastive learning, implicit aspect detection, pyramid pooling, transformer

## Abstract

Implicit aspect detection aims to identify aspect categories that are not explicitly mentioned in text, but existing models struggle with four persistent challenges: aspect ambiguity, where multiple latent aspects are implied by the same expression, data imbalance and sparsity of implicit cues, contextual noise and syntactic variability in unstructured user reviews, and aspect drift, where the relevance of implicit cues changes across sentences or domains. To address these issues, this paper proposes the Transformer-Enhanced Graph Aspect Analyzer (TEGAA), a unified framework that tightly integrates dynamic expert routing, semantic representation refinement, and hierarchical graph reasoning. First, a Dynamic Expert Transformer (DET) equipped with a Dynamic Adaptive Expert Engine (DAEE) mitigates syntactic complexity and contextual noise by dynamically routing tokens to specialized expert sub-networks based on contextual and syntactic–semantic cues, enabling robust feature extraction for ambiguous implicit expressions. Second, Semantic Contrastive Learning (SCL) directly addresses data imbalance and weak implicit signals by enforcing semantic coherence among contextually related samples while increasing separability from irrelevant ones, thereby improving discriminability of sparse implicit aspect cues. Third, implicit aspect ambiguity and aspect drift are handled through a Graph-Enhanced Hierarchical Aspect Detector (GE-HAD), which models word- and sentence-level dependencies via context-aware graph attention. The incorporation of Attention Sinks prevents dominant but irrelevant tokens from overshadowing subtle implicit cues, while Pyramid Pooling aggregates multi-scale contextual information to stabilize aspect inference across varying linguistic scopes. Finally, an iterative feedback loop aligns graph-level reasoning with transformer-level expert routing, enabling adaptive refinement of aspect representations. Experiments on three benchmark datasets—Mobile Reviews, SemEval14, and Sentihood—demonstrate that TEGAA consistently outperforms state-of-the-art methods, achieving F1-scores above 0.88, precision above 0.89, recall above 0.87, accuracy exceeding 89%, and AUC values above 0.89. These results confirm TEGAA’s effectiveness in resolving implicit aspect ambiguity, handling noisy and imbalanced data, and maintaining robust performance across domains.

## 1 Introduction

Implicit aspect detection in NLP finds the aspects that are implicit or hidden in the text instead of being explicitly stated. This is crucial for applications such as sentiment analysis, aspect-based opinion mining, and customer feedback analysis, were users often express opinions indirectly. These implicit aspects can help businesses uncover hidden product strengths, leading to improved development strategies and more targeted marketing efforts (Soni and Rambola, 2022; Maitama et al., 2020). Global sentiment analysis market is believed to reach billion dollars through the year 2030 and is driven by need of such fine customer understanding. Studies have shown how advanced text analytics, also with implicit aspect detection, helps a company increase customer satisfaction and retention (Ganganwar and Rajalakshmi, 2019; Al-Janabi et al., 2022). Implicit aspect detection in such sectors as automotive and social media monitoring has helped brands to make product features better and hidden sentiments more visible, leading to a more effective engagement and management (Truşcǎ and Frasincar, 2023; Zhang et al., 2024).

Implicit aspect detection has progressed from rule-based and traditional machine learning to more advanced deep learning and graph-based approaches (Chauhan et al., 2023; Do et al., 2019). Early approaches depended on manual rules and sentiment lexicons, which are limited by predefined patterns. SVMs and CRFs machine learning models have been able to improve the feature learning of labelled data but failed in coping with language complexity. Recently, with the advent of deep learning, CNNs, RNNs and later transformers like BERT, have helped in contextual embeddings to better detect implicit aspects (Le Thi et al., 2023; Liu et al., 2020). Recently, Graph Neural Networks have been applied to further boost this area by capturing the relational contexts between words and entities that greatly enhance accuracy and robustness in implicit aspect detection (Zhu et al., 2022; Yusuf et al., 2024; Sarno et al., 2024). Explicit aspect detection involves aspects that are directly mentioned, such as “The battery life of this phone is excellent,” where battery life is explicitly stated. In contrast, implicit aspect detection requires inferring aspects from context, as in “I had to charge the phone twice in 1 day,” which implies battery life without explicit mention. Similarly, “The room was so cold that I needed a jacket” implicitly refers to air conditioning, and “It takes forever to get your food here” implies service speed. These examples illustrate the complexity of implicit aspect detection and motivate the need for models like TEGAA that capture latent semantic and contextual cues beyond surface-level keywords.

With an organization seeking deeper insights from an abundance of unstructured text data, implicit aspect detection becomes more crucial. Although great strides have been made so far, there are a few key challenges that call for research. One is the challenge of imbalance in datasets wherein some aspects may be less represented, resulting in biased predictions by the models (Phan et al., 2023; Xu et al., 2023). In addition, the tremendous computation cost of models such as Transformers and GNNs is also expensive, which will be too costly for real-time applications. The rise in fake data and manipulated information has made it challenging to detect the true implicit attributes; hence, models are required that can differentiate between valid and invalid information (Khemani et al., 2024; An et al., 2022). In addition, the complexity in the syntax and grammar of a natural language makes it hard as models have to learn how to interpret and process widely spread linguistic structures in order to detect implicit elements accurately (Soni and Rambola, 2022; An et al., 2022).

### 1.1 Problem statement

Implicit aspect detection in natural language processing remains challenging due to multiple intertwined factors. First, data imbalance and sparsity of implicit cues cause implicit aspects to appear infrequently and be overshadowed by explicit or sentiment-dominant expressions. Second, aspect ambiguity and aspect drift arise when the same linguistic patterns imply different latent aspects depending on context, sentence structure, or domain, leading to inconsistent predictions. Third, contextual noise and syntactic/grammatical complexity, including informal language, noisy or manipulated text, and diverse sentence constructions, hinder robust feature extraction and reliable inference. Addressing these challenges requires a scalable and adaptive framework capable of capturing nuanced semantic relationships while remaining robust to noise and contextual variation (Ocampo et al., 2023; Das and Singh, 2023). The contributions of the proposed model are given below,

- *Dynamic Feature Extraction with DET:* The Dynamic Expert Transformer (DET), enhanced by the Dynamic Adaptive Expert Engine (DAEE) with a Contextual Expert Router (CER) and Adaptive Syntactic-Semantic Router (ASSR), introduces a novel dynamic routing mechanism. Inputs are adaptively assigned to specialized expert sub-networks, enabling richer syntactic and semantic embeddings than standard transformer approaches.
- *Semantic Contrastive Learning (SCL):* Semantic Contrastive Learning refines embeddings by explicitly leveraging semantic relationships. This innovative contrastive framework enhances feature discriminability by aligning semantically related samples and separating unrelated ones, capturing subtle contextual variations that conventional embedding refinement methods often miss.
- *Hierarchical Aspect Detection via GE-HAD:* The Graph-Enhanced Hierarchical Aspect Detector (GE-HAD) constructs multi-level hierarchical context graphs, integrating word-level and sentence-level nodes. This novel hierarchical attention mechanism captures rich multi-granular contextual relationships, enabling more precise detection of implicit aspects compared to existing graph-based methods.
- *Advanced Graph Attention Mechanisms:* The Context-Aware Graph Attention mechanism, combined with Attention Sinks and Pyramid Pooling, aggregates features across multiple scales. This enables selective focus on task-relevant nodes while preserving fine-grained semantic information, surpassing traditional graph attention approaches in capturing nuanced implicit cues.

The paper contains following sections, with section 2 giving a detailed literature review of different studies done for implicit aspect detection, section 3 giving a thorough analysis of the proposed methodology, section 4 analyzing the performance of the proposed model along with quantitative, visual and comparative analysis, section 5 discussing the different advantages of the proposed model with limitations and section 6 concluding the paper with the future prospects of the study.

## 2 Related works

Recent advancements in implicit aspect detection in text streams have explored various innovative approaches, targeting applications like emotion detection, sentiment analysis, and implicit aspect identification. These works collectively contribute to addressing the challenges in extracting and analyzing contextual features from text data.

Ouyang et al. (2024) proposed a word-level relational hypergraph, which could capture diverse syntactic and semantic relationships among aspect words and their contextual terms. To fully exploit this hypergraph, they proposed an Aspect-Specific Hypergraph Attention Network (ASHGAT), which optimizes the attention of the hypergraph by a syntactic distance-based weight distribution mechanism. Still, the model’s quality is highly dependent on syntactic distance calculations, limiting its performance in ambiguous or diverse context scenarios. Expanding on the syntax and semantics of relationships, Hu et al. (2023) presented the Multi-level Semantic Relation-Enhanced Learning Network, which is essentially a reformulation of traditional aspect-based sentiment analysis (ABSA) as a task of semantic alignment. With integrated word and sentence-level semantic relations, MSRL-Net enhances contextual alignment, although dependence on correct semantic mapping may also limit its performance in the nuanced contexts, much like the dependency issues that surface in Ouyang et al.’s work.

To alleviate the weakness associated with traditional similarity computation methods, Benarafa et al. (2023) modified the KNN algorithm by refining its distance computation mechanism to enhance its ability to avoid overfitting and underfitting, two common weaknesses in implicit aspect detection. While successful, their performance is contingent on the selection of distance metrics, which mirrors some of the issues pointed out by previous studies, such as ASHGAT and MSRL-Net, in terms of variability within the dataset. On the other hand, Murtadha et al. (2024) focused on using auxiliary sentence generation to simultaneously classify aspects and perform sentiment analysis. The approach used a BERT-based framework fine-tuned for these tasks. Their approach improves aspect-specific representation learning by reacting to seed semantic distributions within the embedding space. However, similar to ASHGAT and MSRL-Net, their model relies on the quality of these auxiliary sentences, parallel to the reliance on input accuracy.

Focusing on richer contextual embeddings, Liu and Shen (2023) developed the Information-Augmented Neural Network (IANN), incorporating the Multiple Convolution with Recurrence Network (MCRN) to integrate contextual information dynamically. The introduction of the Aspect Outside (AO) tagging scheme provides a simplified yet effective tagging methodology for implicit aspect tagging. However, its success is influenced by the complexity of contextual data integration, as seen in Murtadha et al.’s framework. Extending this focus on contextual understanding, Chouikhi et al. (2023) applied transfer learning for Aspect Term Extraction and Aspect Polarization Detection in Arabic, utilizing pre-trained BERT models tailored for the language. Their comparative analysis of various BERT implementations highlights the sensitivity of performance to model configuration, mirroring the configuration-specific challenges seen in other works, such as IANN and MSRL-Net.

By collectively addressing syntactic dependencies, semantic alignment, distance metrics, auxiliary data generation, contextual embeddings, and transfer learning, these related works demonstrate complementary strengths and shared limitations. These insights underline the importance of integrating robust feature extraction, semantic refinement, and adaptive learning mechanisms to overcome challenges in aspect detection, paving the way for advanced frameworks that build upon these contributions.

The literature on implicit aspect detection reveals several recurring limitations that motivate the design of TEGAA. Models such as ASHGAT (Ouyang et al., 2024) and MSRL-Net (Hu et al., 2023) rely heavily on explicit syntactic distances or predefined semantic mappings, which restrict their ability to generalize when sentence structures vary or when implicit relationships are not explicitly encoded. In contrast, TEGAA avoids rigid linguistic assumptions by dynamically learning contextual dependencies through adaptive expert routing and graph-based relational modeling. Distance-based and metric-dependent approaches, including enhanced KNN (Benarafa et al., 2023) and the framework of Murtadha et al. (2024), further suffer from sensitivity to predefined similarity measures and the quality of auxiliary data, limiting robustness under noisy or manipulated inputs; this limitation is addressed in TEGAA through DET with DAEE, which suppresses noise by selectively activating task-relevant expert sub-networks. Contextual embedding models such as IANN (Liu and Shen, 2023) and transfer-learning strategies by Chouikhi et al. (2023) improve semantic representation but do not explicitly handle class imbalance, aspect ambiguity, or aspect drift, leading to unstable performance across domains. TEGAA addresses these gaps by integrating Semantic Contrastive Learning to counter data imbalance and sparse implicit cues, and a Graph-Enhanced Hierarchical Aspect Detector to disambiguate latent aspects and maintain stability under shifting contexts. By systematically overcoming the structural rigidity, metric dependence, and partial challenge coverage of prior work, TEGAA provides a unified and robust solution for implicit aspect detection.

## 3 Proposed TEGAA

**Figure 1:**
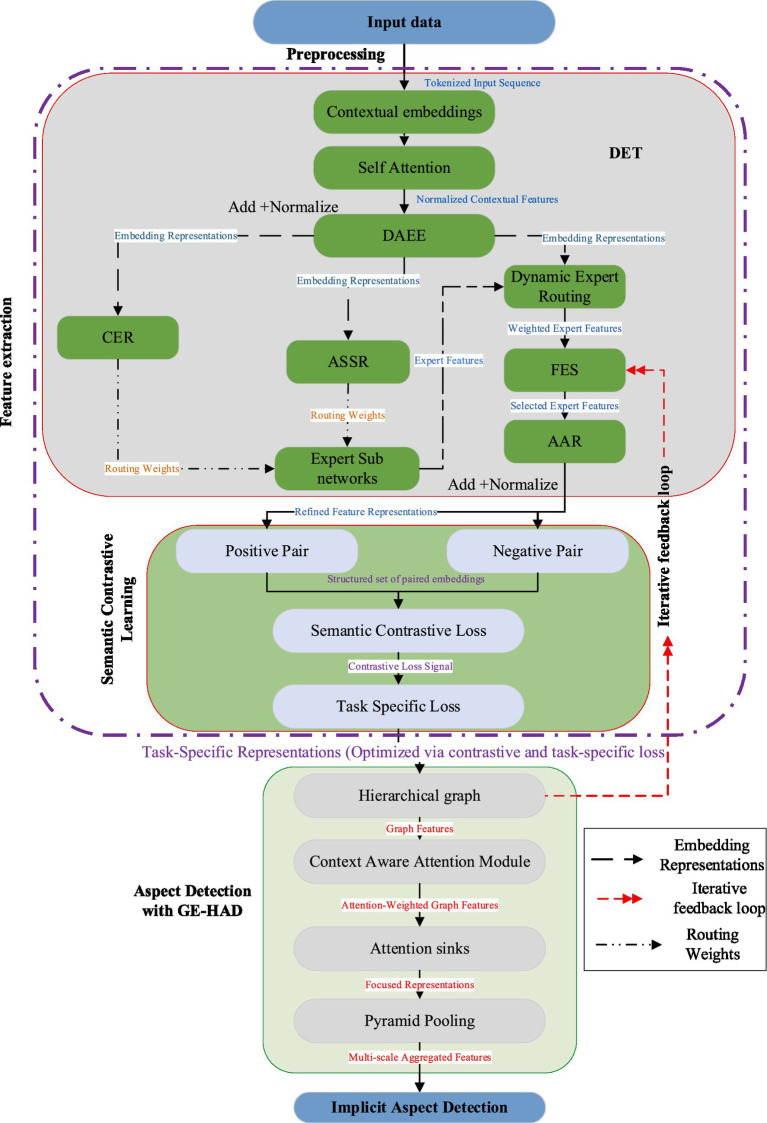
Overall architecture of TEGAA.

The Transformer-Enhanced Graph Aspect Analyzer (TEGAA) is an advanced framework designed for implicit aspect detection in text data, integrating state-of-the-art techniques in both feature extraction and implicit aspect detection. The model leverages the Dynamic Expert Transformer (DET) based on switch transformer (Fedus et al., 2022) with the Dynamic Adaptive Expert Engine (DAEE) to extract rich contextual embeddings from unstructured text. DET uses a custom gating mechanism within DAEE to route embeddings to specialized expert sub-networks, focusing on task-specific syntactic and semantic features. This approach enhances the model’s ability to capture nuanced contextual information and improve feature representations. Followed by feature extraction, Semantic Contrastive Learning refines these embeddings by forming positive pairs from semantically related data points- such as those sharing related topics or sentiments, and negative pairs- using a contrastive loss, to improve the discriminability of the features. In the implicit aspect detection phase, the Graph-Enhanced Hierarchical Aspect Detector (GE-HAD) plays a pivotal role. GE-HAD constructs a multi-level hierarchical graph, integrating nodes at distinct levels for capturing rich contextual relationships. The model employs Context-Aware Graph Attention mechanism to dynamically adjust attention weights based on DET’s embeddings. Attention Sinks (Xiao et al., 2023) and Pyramid Pooling (Elhassan et al., 2024) are incorporated to focus on critical nodes and aggregate features at multiple scales, ensuring effective feature combination and comprehensive implicit aspect detection. TEGAA’s iterative feedback loop, which integrates the hierarchical graph outputs (Yang et al., 2021) to refine DET’s gating decisions and attention patterns, further enhances the model’s performance. This feedback mechanism allows TEGAA to adapt to the nuances of the data, ensuring accurate and contextually relevant implicit aspect detection. The final model is evaluated through different performance measures to validate its effectiveness and accuracy. The overall architecture of the proposed model is illustrated in Figure 1.

In the figure, Add+Normalize refers to adding a residual connection followed by Layer Normalization. This combination helps stabilize training, allowing gradients to flow more effectively and ensuring consistent activation scales across layers. It is commonly used after attention or feed-forward transformations.

### 3.1 Feature extraction with dynamic expert transformer

The Dynamic Expert Transformer (DET) improves feature extraction capabilities through its Dynamic Adaptive Expert Engine (DAEE), the mechanism that captures rich contextual embeddings from unstructured text. DET implements a custom gating mechanism within DAEE, specifically the Contextual Expert Router (CER) and the Adaptive Syntactic-Semantic Router (ASSR), to dynamically route inputs to specialized expert sub-networks based on specific linguistic and semantic features of the task. CER: This guides the embeddings toward the relevant expert based on contextual relevance. ASSR: This prioritizes the sub-networks of the expert toward syntactic and semantic aspects. This targeted routing helps DET to focus on the features such as syntactic dependencies and semantic nuances that are crucial for implicit aspect detection. The Feedback-Driven Expert Selector (FES) also adjusts the dynamic expert selection with the feedback from Graph-Enhanced Hierarchical Aspect Detector (GE-HAD), adapting to the dynamic interplay. This iterative feedback loop, mediated by the Aspect-Aware Attention Refiner (AAR), continually refines DET’s gating decisions and attention patterns. This will lead to a more finely attuned feature extraction process to the nuances in the data, which should significantly improve the overall accuracy of implicit aspect detection. DET employs advanced routing, feedback, and attention mechanisms for better representation and capturing of nuanced features in the unstructured text. Key components of this model include extraction of rich contextual, syntactic, and semantic embeddings based on the DAEE engine. In the Dynamic Expert Transformer (DET), the Dynamic Adaptive Expert Engine (DAEE) leverages five expert sub-networks (*K* = 5) to capture diverse linguistic and contextual patterns crucial for implicit aspect detection. Each expert specializes in distinct tasks, including syntactic parsing, semantic role labeling, sentiment cue recognition, and discourse-level reasoning, allowing the model to dynamically process complex textual structures. The token embeddings within DET are initialized using BERT-base-uncased, providing rich contextual representations that are subsequently routed through the experts via the Contextual Expert Router (CER) and Adaptive Syntactic-Semantic Router (ASSR). This design enables adaptive feature extraction, where each expert focuses on task-relevant information, and the system iteratively refines representations through feedback from the Graph-Enhanced Hierarchical Aspect Detector (GE-HAD), ensuring robust handling of ambiguous, sparse, or contextually nuanced implicit aspects.

To adapt the original Switch Transformer for implicit aspect modeling, the proposed Dynamic Expert Transformer (DET) incorporates several architectural modifications that extend beyond standard sparse MoE routing. First, the fixed top-1 gating mechanism is replaced with a context-aware multi-expert router that conditions gating probabilities on both token embeddings and auxiliary syntactic–semantic cues, enabling more fine-grained expert selection for aspect-related patterns. Second, DET integrates a Dynamic Adaptive Expert Ensemble (DAEE), allowing the routing distribution to be iteratively refined using feedback signals produced by the GE-HAD graph module, rather than relying solely on the initial token-level gating decision. Third, each expert is augmented with aspect-sensitive normalization and lightweight residual adapters, encouraging specialization toward distinct implicit aspect cues. Finally, DET introduces a feedback-compatible gating update rule, ensuring that expert assignments can be adjusted across iterations based on graph-level contextual information. These modifications collectively differentiate DET from the original Switch Transformer and tailor it specifically for robust and adaptive implicit aspect detection.

#### 3.1.1 Dynamic adaptive expert engine

The Dynamic Adaptive Expert Engine (DAEE) serves as the coordination core of the Dynamic Expert Transformer, ensuring that feature extraction is contextually informed, syntactically aware, and dynamically refined through feedback. It operates through four interconnected components that work in a sequential yet iterative manner. The Contextual Expert Router (CER) initiates routing by analyzing discourse-level cues and assigning tokens to specialized expert sub-networks based on contextual relevance. The Adaptive Syntactic-Semantic Router (ASSR) refines these assignments by integrating syntactic dependencies and semantic embeddings, enabling linguistically coherent expert selection. The Feedback-Driven Expert Selector (FES) then incorporates performance signals from the downstream GE-HAD module, dynamically correcting misrouted or ambiguous tokens through iterative updates. Finally, the Aspect-Aware Attention Refiner (AAR) adjusts expert-level attention weights to prioritize tokens carrying implicit aspect cues. Together, these four components form a high-level, closed-loop mechanism that organizes how expert networks communicate, refine one another’s outputs, and continuously improve representation quality-providing the conceptual structure needed to understand the mathematical details that follow.

In practical processing, DAEE dynamically selects the most relevant expert networks through a gating mechanism that computes soft routing probabilities for each token. After the input text is embedded into high-dimensional vectors, each expert-such as one specializing in syntactic patterns or another in semantic relations-receives tokens according to these routing probabilities. CER first directs key contextual elements (e.g., “lasts” and “all day” in “The phone lasts all day”) toward an expert tuned for battery-related semantics. ASSR then reinforces or corrects these assignments by combining dependency relations (linking “lasts” to “phone”) with semantic embeddings, reducing misrouting errors common in simpler models. FES further adjusts the routing based on GE-HAD’s hierarchical graph outputs, enabling the system to distinguish subtle cases such as “vibrant screen” versus “drains quickly,” and AAR enhances implicit cue recognition by amplifying aspect-specific signals (e.g., “long-lasting” → battery life). Through this coordinated interplay-contextual routing, syntactic–semantic refinement, feedback-driven correction, and attention adjustment-DAEE provides an intuitive, high-level overview of how expert networks work together before the formal equations in the methodology section. This coordinated interplay enables the DAEE to adaptively process text, achieving semantic coherence and discriminability. By integrating contextual analysis, syntactic-semantic routing, feedback-driven adjustments, and attention refinement, the DAEE ensures robust feature extraction, setting the stage for the detailed mathematical formulations in Equations 3–11.

Let the input embedding *x∈R^d^*, where *d* is the dimensionality. The DAEE defines *K* expert networks {*E*_1_, *E*_2_, …, *E_K_*}, each with distinct specializations. The gating mechanism computes a distribution over experts as given in Equation 1:


$$g(x)=\sigma (\frac{W_{g}x+b_{g}}{\tau }),g(x)\in {\mathbb{R}}^{K}$$ (1)

Where, *σ* represents the softmax function, providing a probability distribution over *K* experts, *W_g_∈R^K × d^* and *b_g_∈R^K^* are trainable parameters, *τ > 0* is a temperature parameter that controls the sharpness of the distribution. Lower *τ* increases confidence in the gating decision, while higher *τ* distributes probabilities more evenly. As given in Equation 2, the DAEE output *Y_D_* combines the outputs of selected experts weighted by *g(x)*,


$$Y_{D}=\sum_{i=1}^{K}g_{i}(x).E_{i}(x)$$ (2)

Where, *g_i_*(*x*) is the probability assigned to expert *i*, and *E_i_*(*x*) is the output of expert *i*. DAEE ensures adaptive feature extraction by dynamically routing inputs to the most relevant experts, leveraging their specialized knowledge. For the sentence “The quick brown fox jumps over the lazy dog,” the system might route to Expert 1 for analyzing syntactic relations, while Expert 2 may handle semantic information like action or motion (e.g., “jumps over”). The final output is a combination of these experts weighted by their relevance.

#### 3.1.2 Contextual expert router

The Contextual Expert Router (CER) directs embeddings to experts based on contextual features, such as sentence structure, discourse, and topic relevance. It evaluates the relevance of an input *x* by considering its contextual embedding *h_c_*(*x*), computed using a context encoder from the switch-based transformer. For example, the CER might assign higher importance to experts that understand action-related words in the context of a motion task.


$$h_{c}(x)=En_{\text{cont}}(x)$$ (3)

Where, *En_cont_* is the context encoder. The routing probabilities are then determined as,


$$r_{\text{cont}}(x)=\sigma (\frac{W_{\text{cont}}h_{c}(x)+b_{\text{cont}}}{\tau }),r_{\text{cont}}(x)\in {\mathbb{R}}^{K}$$ (4)

Where, *h_c_(x)∈R^m^* is the contextual embedding, and *m* is the embedding dimension, *K* represents the number of expert networks, *K* is the dimensionality of the routing probability vector, *W*_cont_∈R^*K* × *m*^ and *b_cont_∈R^K^* are trainable weights for the contextual router. The CER output combines expert outputs, weighted by *r_cont_(x)*,


$$Y_{\mathrm{CER}}=\sum_{i=1}^{K}r_{\text{cont},i}(x).E_{i}(x)$$ (5)

Where, $r_{\text{cont},i}(x)$ is the probability assigned to the *i-th* expert based on the context of the input *x*. CER uses contextual embeddings to prioritize experts, enabling the system to focus on topic-specific or discourse-related features.

#### 3.1.3 Adaptive syntactic-semantic router

The Adaptive Syntactic-Semantic Router (ASSR) routes inputs based on syntactic and semantic relevance. For example, the sentence “The quick brown fox jumps over the lazy dog” has clear syntactic structures such as subject, verb, and object, and semantic relationships such as the action of jumping. It uses two embedding functions:

- *Syntactic embedding h_s_(x)*, derived from dependency parsing: *h_s_​(x) = Parsers_yntactic_(x)*
- *Semantic embedding h_sem_(x)*, derived from pretrained semantic model: *h_sem_(x) = Encoder_semantic_(x)*

The two types of embedding are combined together. The combined embedding is passed through a final gating function to select the most appropriate experts for syntactic or semantic tasks. For example, if the goal is to understand the action (“jumps”), syntactic experts may focus on verb phrases, while semantic experts may focus on the meaning of “jump” in the context of physical motion. The final gating mechanism is given as,


$$\begin{array}{l} h_{\mathrm{syn}-\mathrm{sem}}(x)=[h_{s}(x);h_{\mathrm{sem}}(x)],\;\\ \;r_{\mathrm{syn}-\mathrm{sem}}(x)=\sigma (\frac{W_{\mathrm{syn}-\mathrm{sem}}h_{\mathrm{syn}-\mathrm{sem}}(x)+b_{\mathrm{syn}-\mathrm{sem}}}{\tau })\; \end{array}$$ (6)

Where, *h_syn-sem​_(x)∈R^p + q^* combines syntactic (*p*-dimensional) and semantic (*q*-dimensional) features, *W_syn-sem_∈R^K × (p + q)^*, *h*_syn-sem_∈R*^K^* are trainable parameters and $\left[h_{s}(x);h_{\mathrm{sem}}(x)\right]$ represents the concatenation of the two embeddings. The output of ASSR is expressed as,


$$Y_{\text{ASSR}}=\sum_{i=1}^{K}r_{\mathrm{syn}-\mathrm{sem},i}(x).E_{i}(x)$$ (7)

ASSR ensures experts are selected to handle specific syntactic structures (e.g., clauses) or semantic relationships (e.g., entailment, similarity).

#### 3.1.4 Feedback-driven expert selector

The Feedback-Driven Expert Selector (FES) refines expert selection based on feedback from downstream tasks, such as implicit aspect detection. Once the initial expert outputs are produced, the model evaluates their performance and adjusts future expert selection using the feedback signal *F(y)*. This signal is derived from a Multi-Layer Perceptron (MLP) that processes the output of the Graph-Enhanced Hierarchical Aspect Detector (GE-HAD). The feedback signal *F(y)* adjusts the gating scores,


$$g_{\text{updated}}(x)=\lambda .g(x)+(1-\lambda ).F(y)$$ (8)

Where, *F(y) = MLP_feedback_(Y_GE-HAD_)*, an MLP mapping the downstream output *Y_GE-HAD_* to a feedback signal and *λ* is a weighting factor for the original gating scores. The final output is updated based on the refined gating scores:


$$Y_{\mathrm{FES}}=\sum_{i=1}^{K}g_{\text{updated},i}(x).E_{i}(x)$$ (9)

For instance, if the implicit aspect detection task is focused on identifying aspects like motion or action, the FES will dynamically adjust expert selection, emphasizing experts that have proven more accurate at detecting action-related features.

#### 3.1.5 Aspect-aware attention refiner

The AAR refines the attention mechanism using aspect-specific information. The system computes the attention weights for each expert output and refines them based on aspect-specific information. Given *Y_FES_*, the attention weight matrix is computed as:


$$A_{\text{refined}}=\text{softmax}(\frac{(QW_{Q}){\left(KW_{K}\right)}^{T}}{\sqrt{d_{k}}}+b_{\mathrm{AAR}})$$ (10)

Where, *Q*, *K* are queries and keys derived from *Y_FES_, W_Q_*, *W_K_* are weight matrices, *d_k_* represents the dimension of the key vectors in the attention mechanism and *b*_AAR_ is a bias term. The refined embedding is expressed as,


$$Y_{\mathrm{AAR}}=A_{\text{refined}}VW_{V}$$ (11)

Where, *V* represents values (e.g., expert outputs) and *W_v_* is a trainable weight matrix associated with the values, responsible for transforming the output representation after applying attention. AAR focuses attention on relevant aspects, enhancing DET’s ability to emphasize critical features. The Dynamic Expert Transformer (DET) is a robust framework for extracting complex contextual, syntactic, and semantic features. Its adaptive routing and feedback-driven refinement, significantly improve feature extraction for tasks like implicit aspect detection, sentiment analysis, and text classification. The advanced mathematical formulations ensure precise expert selection and robust hierarchical modeling.

### 3.2 Semantic contrastive learning for enhanced feature extraction

Semantic Contrastive Learning (SCL) enhances feature extraction by refining the representations generated by the Dynamic Expert Transformer (DET) in the Transformer-Enhanced Graph Aspect Analyzer (TEGAA) model. It involves preparing sets of positive and negative sample pairs based on semantic relationships within the data, where semantically similar instances form positive pairs, and dissimilar ones constitute negative pairs. Applying a contrastive loss function maximizes similarity between positive pairs while minimizing it for negative pairs, thereby boosting feature discriminability across contextual variations. This process captures subtle differences in representations, enabling DET to produce more accurate and meaningful embeddings, which strengthens the feature extraction mechanism for distinguishing between similar and dissimilar implicit aspects, ultimately improving TEGAA’s performance in detecting and analyzing nuanced text aspects. Semantic contrastive learning refers to the model’s ability to capture and analyze contextual shifts, patterns, or relationships within the data, such as identifying changes in sentiment or aspect relevance, maintaining coherence across related contexts, or distinguishing unrelated content. This approach supports tasks like tracking evolving trends or understanding contextual dependencies in dynamic text, enhancing TEGAA’s adaptability.

SCL integrates semantic dynamics into the DET feature extraction pipeline, boosting its ability to detect evolving and subtle differences between data representations. By constructing positive and negative sample pairs, SCL employs an advanced contrastive loss to compel DET to generate more contextually coherent and discriminative embeddings, making it well-suited for nuanced implicit aspect detection and analysis. This process enhances the model’s capacity to differentiate subtle variations in input text, particularly when identifying implicit aspects, by focusing on semantic relationships- whether across related sentences or conversational flows- thus capturing essential contextual cues. The contrastive learning approach ensures that embeddings retain critical contextual information, enabling TEGAA to effectively analyze nuances in sentiment or aspect shifts, thereby improving its overall performance in identifying implicit aspects across datasets like mobile reviews, SemEval14 and Sentihood.

In Semantic Contrastive Learning (SCL), positive and negative pairs are carefully constructed to enhance the discriminability of contextual embeddings and improve the model’s ability to capture subtle semantic nuances. Positive pairs consist of embeddings from contextually related tokens or sentences, such as words appearing in the same semantic context or sentences discussing the same aspect, while negative pairs are drawn from unrelated or semantically distant tokens and sentences. A semantic offset is defined as the vector difference between embeddings of semantically related samples, representing meaningful contextual variation in the embedding space. This offset is used to guide the contrastive loss, encouraging embeddings of related samples to be pulled closer together while pushing unrelated samples farther apart.

The use of semantic offsets allows the model to explicitly encode fine-grained relationships between tokens and sentences, ensuring that subtle implicit cues-such as modifiers, sentiment-laden phrases, or context-dependent expressions—are more effectively captured. By incorporating both token-level and sentence-level information, SCL refines the DET-generated embeddings across multiple levels of granularity. This not only improves feature separability but also enhances the overall robustness of the model against noisy or ambiguous inputs, ultimately leading to more accurate implicit aspect detection.

#### 3.2.1 Pair construction with semantic dynamics

The first part of Semantic Contrastive Learning is building positive and negative pairs out of the sequence data. These pairs give the needed contrast to improve the latent representations so that there is no overlap in the embeddings of semantically irrelevant points. This systematic pairing captures the semantic coherence and ensures separability across different text sequences.

- *Positive pairs*: Composed of embeddings from semantically related data points, ensuring coherence across contextually similar content, such as embeddings from text with shared topics or sentiments. For example, embeddings (*z_t_​, z_t + Δ_*​), where *Δ* is a small semantic offset.
- *Negative pairs*: Include embeddings from semantically unrelated data points, introducing diversity and enforcing discriminability, such as embeddings from text with dissimilar topics or sentiments. For example, embeddings (*z_t_*, *z*_*t* + Δ*d*_), where *Δ_d_ ≫ Δ*.

Let *x_t_* represent data in input text sequence. DET processes this through its Dynamic Adaptive Expert Engine (DAEE) to produce embeddings which is expressed as given in Equation 12,


$$z_{t}=f_{\mathrm{DET}}(x_{t})$$ (12)

Where, *f_DET_* encapsulates DET’s expert routing and attention mechanisms. The dataset *D* is split into positive and negative pairs as given in Equations 13,14 respectively:


$$\text{Positive pairs}:P=\{(z_{t},z_{t+\Delta })|\Delta \in [0,\Delta_{\max}[]]\{\}\}$$ (13)

$$\text{Negative pairs}:N=\{(z_{t},z_{t+\Delta_{d}})|\Delta_{d}>\Delta_{\max}\{\}\}$$ (14)

Here, *Δ_max_* represents the threshold for semantic similarity. The semantic contrastive loss as calculated in Equation 15 aims to maximize similarity for positive pairs while minimizing it for negative pairs, creating discriminative embeddings sensitive to semantic relationships.


$$L_{\mathrm{SCL}}=-\frac{1}{|D|}\sum_{t\in D}\log\frac{e\mathrm{xp}(\mathrm{sim}(z_{t,}z_{t+\Delta })/\tau )}{\begin{array}{l} \exp(\mathrm{sim}(z_{t},z_{t+\Delta })/\tau )+\\ \sum_{z_{t+\Delta_{d}\in N}}\exp(\mathrm{sim}(z_{t},z_{t+\Delta_{d}})/\tau ) \end{array}}$$ (15)

Here, sim(*z_i_*, *z_j_*) is the Similarity function, typically cosine similarity given as $\mathrm{sim}(z_{i},z_{j})=\frac{z_{i}.z_{j}}{\left\|z_{i}\|\;\|z_{j}\right\|}$ , *τ* is the temperature scaling factor, controlling the sharpness of similarity distribution, *D* is the entire dataset of embeddings and *ǀDǀ* is the total number of samples. This loss function is a normalized temperature-scaled cross-entropy loss designed to optimize the alignment of positive embeddings while encouraging diversity between negative pairs. SCL refines embeddings through two crucial mechanisms: semantic coherence and semantic discriminability. Semantic coherence ensures that embeddings remain stable for contextually related points by minimizing the distance between positive pairs, capturing gradual transitions in features. This is particularly effective in datasets where subtle semantic variations represent significant changes.

(a) Semantic coherence

- For positive pairs *(z_t_, z_t + Δ_)*, SCL ensures embeddings remain consistent across semantic shifts. By minimizing intra-pair distance given as $\min∥z_{t}-z_{t+\Delta }∥{\;}_{2}^{2}$ . DET learns to capture gradual transitions in feature representations.

(b) Semantic discriminability

- For negative pairs (*z_t_*, *z*_*t* + Δ*d*_), SCL enforces a larger margin of $\max∥z_{t}-z_{t+\mathrm{\Delta d}}∥{\;}_{2}^{2}$ . This separation prevents DET from conflating semantically dissimilar aspects.

The combination of these objectives helps DET distinguish fine-grained semantic variations and contextually irrelevant information. To enhance the learning process, SCL incorporates feedback-driven adaptation. This involves evaluating the quality of embeddings through performance metrics obtained from downstream tasks.

1. *Feedback Generation*: The model evaluates the quality of embeddings via downstream tasks, such as implicit aspect detection or classification.
2. *Embedding Adjustment*: Using the feedback, the Feedback-Driven Expert Selector (FES) re-routes ambiguous cases to specialized experts within DET, ensuring embeddings are further refined for challenging instances.

This adaptive feedback loop reduces the residual errors and improves the discriminability of features. The improved embeddings obtained through SCL show the following advanced properties: Evolving semantic patterns are captured due to semantic adaptation, making the model invariant to semantic variations in the data. Noise robustness is obtained since spurious correlations are eliminated, due to meaningful separations of pairs in the embedding space, by the constraints put on pairs. Finally, semantic precision is maintained by aligning the embeddings for semantically similar instances, even in the presence of noise or variability in the input data. These enhancements collectively improve the model’s ability to handle complex data effectively, which contributes to superior task performance.

The SCL loss is integrated with the DET’s task-specific loss to form a unified optimization objective. Combining the SCL loss, which focuses on refining semantic relationships, with the task-specific loss that optimizes the model for its target application ensures that the embeddings are fine-tuned for both capturing contextual similarities and meeting task-specific requirements during the joint training process. DET and SCL collaborate through iterative updates of model parameters, driven by gradients from the joint loss, to achieve robust learning of semantic representations and improved task outcomes as given in Equation 16.


$$L_{\text{total}}=\alpha L_{\mathrm{SCL}}+\beta L_{\mathrm{DET}}$$ (16)

Where, *α* and *β* are weights balancing semantic contrastive learning and DET’s primary training objectives and *L_DET_* denotes Task-specific loss (e.g., cross-entropy for classification). This joint optimization ensures that SCL complements DET’s existing strengths, yielding a robust feature extraction mechanism tailored for dynamic textual data.

### 3.3 Aspect detection with graph-enhanced hierarchical aspect detector

GE-HAD advances implicit aspect detection through multi-level hierarchical context graphs by constructing and analyzing the comprehensive graph of word-level and sentence-level nodes that captures a rich set of contextual relationships throughout the text. Through the use of DET-provided embeddings, Context-Aware Graph Attention mechanism dynamically adapts the weights of attention to concentrate at different granularities toward relevant features and relationships for the model. Attention Sinks are used to rank and process the most important contextual information so that important things are focused on. Pyramid Pooling is used for effective aggregation of features at different scales, ranging from word-level to sentence-level, in order to capture multi-level context and improve feature representations. This combination of attention mechanisms and pooling strategies enhances the ability of the model to detect nuanced and complex aspects by utilizing structured context information and refined attention patterns, which increases the accuracy and depth of implicit aspect detection. GE-HAD refines implicit aspect detection using a multi-level hierarchical graph structure which is able to integrate word-level and sentence-level contexts. Through the application of Context-Aware Graph Attention (CAGA), Attention Sinks, and Pyramid Pooling, it provides precise contextual modelling and feature refinement for fine-grained implicit aspect detection.

#### 3.3.1 Hierarchical context graph construction

The hierarchical graph *G = (V, E)* plays a vital role in capturing the underlying structure of text data, such as sentences and words, within a multi-level context. The nodes in the graph represent distinct levels of text granularity.

Nodes *V*

- Word-level nodes (*v_w_*) represent individual words. They are crucial for capturing the basic linguistic elements and semantic features of the text.
- Sentence-level nodes (*v_s_*) aggregate word nodes within sentences. The sentence nodes encode contextual relationships between words, providing a higher level of abstraction.

Edges *E*

- Intra-level edges (*E_intra_*): These edges capture relationships within the same level. For example, word co-occurrence or syntactic dependencies like subject-verb-object relationships are captured here. These connections are vital for understanding local linguistic structures.
- Inter-level edges (*E_inter_*): These edges link nodes across levels. For instance, word nodes are connected to sentence nodes. These inter-level connections help build a global understanding of the text by linking detailed word-level information to higher-level contextual structures.

The adjacency matrix *A* encodes these relationships, where edge weights *w_ij_* represent semantic similarity or syntactic relevance between nodes.


$$A_{\mathrm{ij}}=\{\begin{array}{c} w_{\mathrm{ij}},\;\;\text{if}(v_{i},v_{j})\in \varepsilon \\ 0,\;\text{otherwise} \end{array}$$ (17)

Where, *w_ij_* is the edge weight calculated based on semantic similarity or syntactic relevance. It is expressed as,


$$W_{\mathrm{ij}}=\exp(-\frac{{\left\|z_{i}-z_{j}\right\|}_{2}^{2}}{\sigma^{2}})$$ (18)

Where, *z_i_​, z_j_* denote feature embeddings of nodes *v_i_, v_j_* and *σ* is the scaling factor controlling sensitivity to differences. To understand the global structure of the graph, the Graph Laplacian *(L)* is computed as given in Equation 19, which helps smooth node representations across the graph, ensuring that closely related nodes have similar embeddings.


$$L=I-D^{\frac{-1}{2}}AD^{\frac{-1}{2}}$$ (19)

Where, *I* represent the identity matrix and *D* is the degree matrix, with *D_ii_ = ∑_j_A_ij_*.

In the Hierarchical Context Graph (HCG), edges are constructed to capture both intra-level (within the same level, e.g., word-to-word or sentence-to-sentence) and inter-level (across levels, e.g., word-to-sentence) dependencies, ensuring comprehensive modeling of contextual relationships. Intra-level edges at the word level are built using dependency parsing tools (such as SpaCy or Stanford CoreNLP) to connect words based on syntactic relations like subject–verb, modifier–noun, and object–verb links, capturing grammatical structure. At the sentence level, intra-level edges are formed using attention-based clustering, where sentence embeddings are computed via the pre-trained transformer, and sentences with cosine similarity above a threshold (e.g., 0.7) are connected, ensuring that semantically related sentences influence each other. Inter-level edges are constructed to link words to the sentences they belong to, as well as to other relevant higher-level constructs. A fixed window size strategy is applied, where each word is connected to neighboring sentences within a context window of ±1 or ±2 sentences, allowing the model to capture short-range discourse dependencies. Additionally, cross-level attention scores from the Context-Aware Graph Attention (CAGA) module are used to weight inter-level edges dynamically, prioritizing words that are semantically or sentimentally significant for implicit aspect detection. This combination of syntactic parsing, attention-based clustering, and window-based connections enables the HCG to represent fine-grained intra-level interactions while maintaining robust inter-level contextual reasoning, which is critical for capturing subtle cues in implicit aspect detection.

Before applying the hierarchical attention mechanism in GE-HAD, all node features in the context graph are initialized using representations generated by the Dynamic Expert Transformer (DET). Each word-level node is assigned its initial feature vector from the DET output corresponding to that token, ensuring that the node embedding already encodes rich syntactic, semantic, and contrastive-learning–refined information. Sentence-level nodes are initialized by computing the mean-pooled embedding of all DET-generated token features within the sentence, allowing each sentence node to capture broader contextual meaning beyond individual words. For graph connectivity, intra-level edge weights are initialized using syntactic dependency strengths or semantic similarity scores between node pairs, while inter-level edge weights are initialized using cosine similarity between word and sentence embeddings. This initialization strategy ensures that both word- and sentence-level nodes begin the GE-HAD process with context-aware, structurally meaningful features, enabling the subsequent attention mechanism to operate on well-formed hierarchical representations.

#### 3.3.2 Context-aware graph attention

The Context-Aware Graph Attention mechanism focuses on refining node representations by incorporating contextual information from neighboring nodes. This is particularly important for text data, as different words or sentences contribute varying levels of importance to the overall meaning. Node representations *z_i_* are iteratively updated to incorporate information from neighbors, weighted by attention coefficients *α_ij_*,


$$z_{i}^{\prime }=\gamma (\sum_{j\in N(i)}\alpha_{\mathrm{ij}}W_{g}z_{j})$$ (20)

Where, *N(i)* represents the Neighbors of node *v_i_*, *W_g_* represents the trainable weight matrix for graph-based transformation and *γ* is the ReLU activation function. Attention coefficients *α_ij_* are computed as,


$$\alpha_{\mathrm{ij}}=\frac{\exp(\phi (z_{i},z_{j}))}{\sum_{k\in N(i)}\exp(\phi (z_{i},z_{k}))}$$ (21)

Where, *ϕ*(*z_i_*​,*z_j_*) is a compatibility function,


$$\phi (z_{i},z_{j})=\psi (a^{T}[W_{g}z_{i}\|W_{g}z_{j}])$$ (22)

Here, *a* is a trainable attention vector, *ψ* represents the leaky ReLU activation function and *||* is the concatenation operator.

#### 3.3.3 Attention sinks

Attention Sinks are special nodes introduced for each level (*v_sink,word_, v_sink,sent_*) to aggregate critical contextual information and prioritize important aspects. These nodes help aggregate the most critical information and allow the model to focus on the most relevant parts of the text. The representation of a sink node *z_sink_* is computed as,


$$z_{\sin k}=\sigma (\sum_{j\in N(\sin k)}\beta_{\sin k,j}W_{s}z_{j})$$ (23)

Where, *N_(sink)_* denotes the Neighboring nodes of the sink, *β*_sink,*j*_ represents the attention weight between the sink and its neighbors, defined similarly to *α_ij_*, and *W_s_* is the Sink-specific weight matrix. By incorporating attention sinks, the model effectively aggregates critical information from multiple text levels (word, sentence) and prioritize aspects that are key for the task, such as sentiment or aspect classification.

In the Graph-Enhanced Hierarchical Aspect Detector (GE-HAD), Attention Sinks represent a pivotal innovation that distinguishes TEGAA from models relying solely on standard attention mechanisms. Conventional approaches—such as the scaled dot-product attention used in Transformers-compute relevance scores between all node pairs, distributing focus broadly across the graph. Under noisy or imbalanced text conditions, this often dilutes sparse but crucial implicit cues, allowing high-frequency or sentiment-heavy tokens to dominate the attention landscape. Attention Sinks overcome this limitation by introducing dedicated nodes at both the word and sentence levels that function as specialized “information hubs.” These sink nodes selectively aggregate contextually important signals from neighboring nodes and assign greater importance to task-relevant features such as implicit aspect cues (e.g., “long-lasting” → battery life), sentiment modifiers, or rare contextual indicators. By concentrating essential semantic evidence and suppressing uninformative dependencies, Attention Sinks offer a targeted refinement layer that standard attention cannot provide.

This behavior is illustrated by the review “The phone lasts all day, but the screen is dull.” In a conventional attention setup, prominent terms like “screen” and “dull,” together with the discourse marker “but,” often attract disproportionately high attention because they are sentiment-heavy and occur frequently in review datasets. This causes the subtle but crucial implicit cue “lasts all day”-which strongly indicates the battery life aspect-to receive comparatively low attention, leading the model to overlook one of the most meaningful signals in the sentence. The Attention Sink mechanism counteracts this effect by inserting a specialized sink node at the sentence level that acts as a semantic collector for low-salience yet task-critical features. Embeddings from tokens such as “lasts” and “all day” are routed into this sink node, which aggregates, normalizes, and amplifies them through learned sink weights *(β_sink, j_)*. Because the sink node functions as a focused reservoir for weak contextual cues, the enhanced representation is pushed back into the graph with a stronger gradient contribution, ensuring that the battery-related meaning remains highly salient even when overshadowed by more dominant tokens. Additionally, irrelevant or noisy words-such as “but,” “the,” or other syntactically necessary yet semantically weak terms-contribute minimally to the sink since the learned weights naturally down-rank them. This selective aggregation not only protects subtle implicit cues from being lost, but also stabilizes attention by reducing overfitting to high-frequency distractors, a common issue in standard attention where such terms can inflate pairwise similarity scores. Furthermore, by acting as a dedicated focal node, the sink helps the model maintain consistency across sentences with similar implicit cues, improving generalization across domains and linguistic variations (e.g., “goes the whole day,” “barely needs charging”). Through this hierarchical refinement, Attention Sinks enable GE-HAD to consistently elevate sparsely distributed but semantically rich signals, substantially enhancing its ability to resolve aspect ambiguity, handle noisy or imbalanced inputs, and detect implicit aspects with higher precision and interpretability.

#### 3.3.4 Pyramid Pooling

The Pyramid Pooling mechanism in the Graph-Enhanced Hierarchical Aspect Detector (GE-HAD) is designed to aggregate contextual information at multiple levels of granularity-word-level and sentence-level-allowing the model to jointly capture fine-grained semantics and broader discourse patterns. At the word level, each node’s embedding is aggregated using attention-weighted mean pooling, where the weights are derived from the Context-Aware Graph Attention (CAGA) coefficients (αᵢⱼ). This ensures that semantically critical words, such as modifiers or implicit aspect indicators, receive higher importance in the pooled representation, while neutral or redundant tokens contribute less. At the sentence level, the model performs context-weighted pooling over the aggregated word-level features within each sentence. Here, inter-level edge weights (wᵢⱼ) from the hierarchical graph are employed to modulate the influence of each word on its corresponding sentence representation, capturing intra-sentence dependencies and inter-sentence coherence. The outputs from both levels are then concatenated and fused through a hierarchical integration layer, forming a composite feature vector that preserves both local lexical nuances and global contextual relationships. This hierarchical fusion mirrors the structure of a pyramid—fine-grained details form the base, while abstracted global semantics form the apex—enabling the model to handle implicit aspect cues that vary in linguistic scope. Unlike conventional pooling operations (e.g., simple mean or max pooling), this attention-guided, multi-scale aggregation adaptively weighs contributions based on contextual relevance, resulting in richer, noise-robust embeddings that significantly enhance implicit aspect detection performance.

Pyramid Pooling enhances the model’s ability to capture hierarchical context by pooling features at multiple scales. This method is effective for text, as it allows the model to learn features at various levels of abstraction, from individual words to different sentences.

- Word-Level Pooling: At the word level, pooling aggregates features based on the individual words in the sentence. This allows the model to focus on the local context of each word.

$$z_{\text{Word}}=\frac{1}{|v_{w}|}\sum_{v_{w}}z_{w}$$ (24)

Where, *Z_w_* denotes the feature representations (or embeddings) for the word-level nodes, capturing the semantic meaning of each word in the sentence and *V_w_* refers to the set of all word-level nodes in the graph, representing the individual words within the sentence.

ii. Sentence-Level Pooling: At the sentence level, pooling aggregates features across sentences. This helps the model capture relationships between sentences and the context they provide for implicit aspect detection.

$$z_{\text{sent}}=\underset{v_{s}}{\max(z_{s})}$$ (25)

Where, *Z_s_* represents the feature embeddings for the sentence-level nodes, which aggregate the word-level features *Z_w_* to capture the overall meaning of each sentence and *V_s_* is the set of all sentence-level nodes, representing the collection of sentences.

The final hierarchical feature vector combines all of these pooled features, allowing the model to incorporate both local and global context into its decision-making process.


$$z_{\text{hier}}=[z_{\text{word}}\|z_{\text{sent}}]$$ (26)

The refined hierarchical feature representation z*_hier_* is used for classification. The implicit aspect prediction is computed as,


$$\hat{Y}=\text{softmax}(W_{\mathrm{cls}}z_{\text{hier}}+b_{\mathrm{cls}})$$ (27)

Where, *W_cls_* is the classification weight matrix and *b_cls_* is the bias term. This final step maps the refined features to the appropriate aspect labels, enabling the model to classify different aspects (e.g., sentiment, product features) in the text. This term enforces smoothness in the node embeddings, ensuring that neighboring nodes have similar representations. It helps avoid overfitting and maintains the coherence of the learned graph structure.


$$L_{\text{graph}}=\sum_{(i,j)\in \varepsilon }{\left\|z_{i}-z_{j}\right\|}_{2}^{2}+\lambda \sum_{i}{\left\|z_{i}\right\|}_{1}$$ (28)

In Equation 28, *ɛ* is the set of edges in the graph, representing the relationships between nodes. *z_i_* is the feature representation (embedding) of node *v_i_*, which corresponds to word-level or sentence-level depending on the context. Similarly, *z_j_* is the feature representation (embedding) of node *v_j_*, representing the features of another node in the graph. The term ${|z_{i}-z_{j}\|}_{2}^{2}$ represents the squared Euclidean distance between the feature vectors *z_i_* and *z_j_*, which ensures that similar nodes, based on their features, have closer embeddings. *λ* is a regularization hyperparameter that controls the strength of the sparsity term, and *ǀǀziǀǀ_1_* denotes the L_1_ norm of the feature vector *z_i_*, which promotes sparsity by penalizing large values in the embeddings. This combination ensures that the model enforces smooth and sparse embeddings, improving both interpretability and computational efficiency. For the aspect classification task, the cross-entropy loss as calculated in Equation 29 is used, which penalizes incorrect predictions. This helps the model learn to predict the correct aspect label for each input.


$$L_{\text{task}}=-\sum_{i=1}^{N}y_{i}{\hat{\log y}}_{i}$$ (29)

As calculated in Equation 30, the total loss is a weighted sum of the graph regularization loss and task-specific loss,


$$L_{\mathrm{GE}-\mathrm{HAD}}=\lambda_{1}L_{\text{graph}}+\lambda_{2}L_{\text{tasks}}$$ (30)

**Figure 2:**
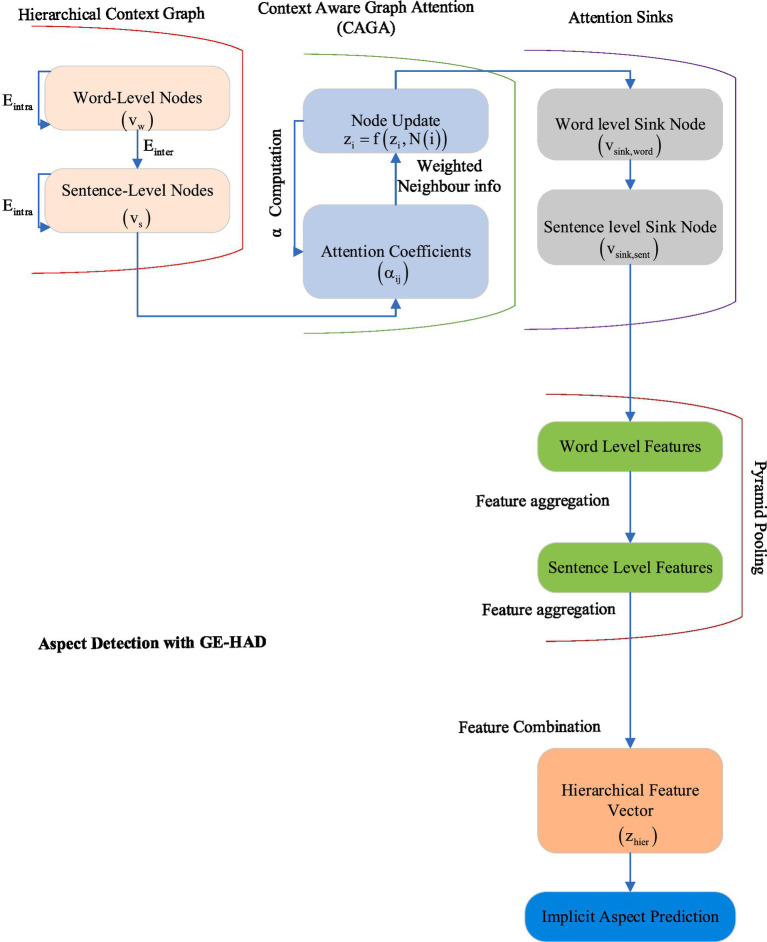
GE-HAD architecture.

This combined loss ensures that the model not only performs well in classifying aspects but also maintains a coherent graph structure that captures the relationships between text elements at different levels. By combining these components-hierarchical context graph construction, graph attention, attention sinks, pyramid pooling, and effective loss functions-the model is able to process and understand text at multiple levels of abstraction, leading to accurate aspect-based sentiment analysis and other related tasks. Figure 2 illustrates the diagram of the proposed GE-HAD module.

The Pyramid Pooling mechanism in the Graph-Enhanced Hierarchical Aspect Detector (GE-HAD) is a hierarchical feature aggregation strategy designed to capture contextual information at multiple levels of granularity, addressing the challenge of detecting implicit aspects in complex, unstructured text. Unlike standard pooling methods that apply uniform aggregation, such as mean or max pooling across all tokens, Pyramid Pooling employs a multi-scale approach through distinct Word-Level Pooling and Sentence-Level Pooling strategies. Word-Level Pooling operates on the feature representations of word nodes, aggregating them using a weighted mean pooling operation in which the weights are derived from attention coefficients. These coefficients prioritize semantically significant words, such as modifiers or implicit aspect indicators (for example, *“long-lasting”* implying battery life in the Mobile Reviews Dataset), ensuring that fine-grained semantic cues critical for implicit aspect detection are preserved. Sentence-Level Pooling, conversely, aggregates word-level features into sentence-level embeddings by applying mean pooling across all word nodes within a sentence, further modulated by inter-level edge weights from the hierarchical graph’s adjacency structure. This process captures broader contextual relationships, such as discourse flow and inter-sentence dependencies, which provide the global context necessary for anchoring implicit aspects within the text.

The outputs from these two pooling levels are combined through concatenation to form the final hierarchical feature vector. This integrated vector brings together both local (word-level) and global (sentence-level) features, which is then passed through a classification layer to predict aspect categories. This multi-scale aggregation is essential because relying solely on word-level pooling may overlook discourse-level context, such as sentiment coherence across sentences, while relying only on sentence-level pooling risks missing subtle implicit signals embedded in individual words. For example, in the Sentihood Dataset, a review stating *“The area feels quiet”* may implicitly reference *“safety,”* a cue that word-level pooling captures through terms like *“quiet,”* while sentence-level pooling ensures the broader context of neighborhood sentiment is considered. This dual-level approach enhances TEGAA’s ability to resolve aspect ambiguity and achieve robust performance.

After pyramid pooling, the model leverages the hierarchical feature representation obtained from the multi-level graph and pyramid pooling. Finally, this aggregated feature vector goes through a classification layer that computes the aspect labels of predictions using a softmax function; in this way, the model correctly classifies implicit aspects based on refined contextual information. The model is strengthened with graph regularization and task-specific loss functions for learning meaningful representations while being smooth and sparse in embeddings. It, therefore, optimizes the loss function that deals both with the detection of relevant aspects and the structural integrity of the graph, hence bettering model performance. Through such sophisticated techniques, the last layer refines implicit aspect detection but ensures that the model is capable of dynamic adaptation toward complex and changing textual contexts. This makes it very effective for nuanced aspect analysis across a range of textual data.

The iterative feedback loop in TEGAA refines the Transformer’s gating decisions by incorporating information from the graph outputs generated by GE-HAD. After GE-HAD computes updated hierarchical node representations, the final graph-level embedding $Y_{\mathrm{GE}-\mathrm{HAD}}$ is passed through a lightweight MLP to produce a feedback signal F(y) , which captures the model’s current understanding of implicit aspect relevance. This signal is used to adjust the DET gating probabilities through an additive refinement term, expressed as $g^{\prime }=\mathrm{\lambda g}(x)+(1-\lambda )F(y)$ , where g(x) is the original expert-routing distribution, F(y) is the feedback-modulated correction, and λ controls the balance between the initial and refined gating decisions. The updated gating distribution $g^{\prime }$ is then normalized via softmax and used to re-route token embeddings across the expert sub-networks in the next iteration. Through this mechanism, ambiguous or weakly represented aspects detected in the graph directly influence expert selection, allowing the system to gradually emphasize experts that better capture implicit cues. This mathematically grounded refinement process ensures tighter alignment between graph-level contextual reasoning and transformer-level feature extraction. Algorithm 1 outlines the sequence of steps used in the training procedure of the proposed TEGAA model.


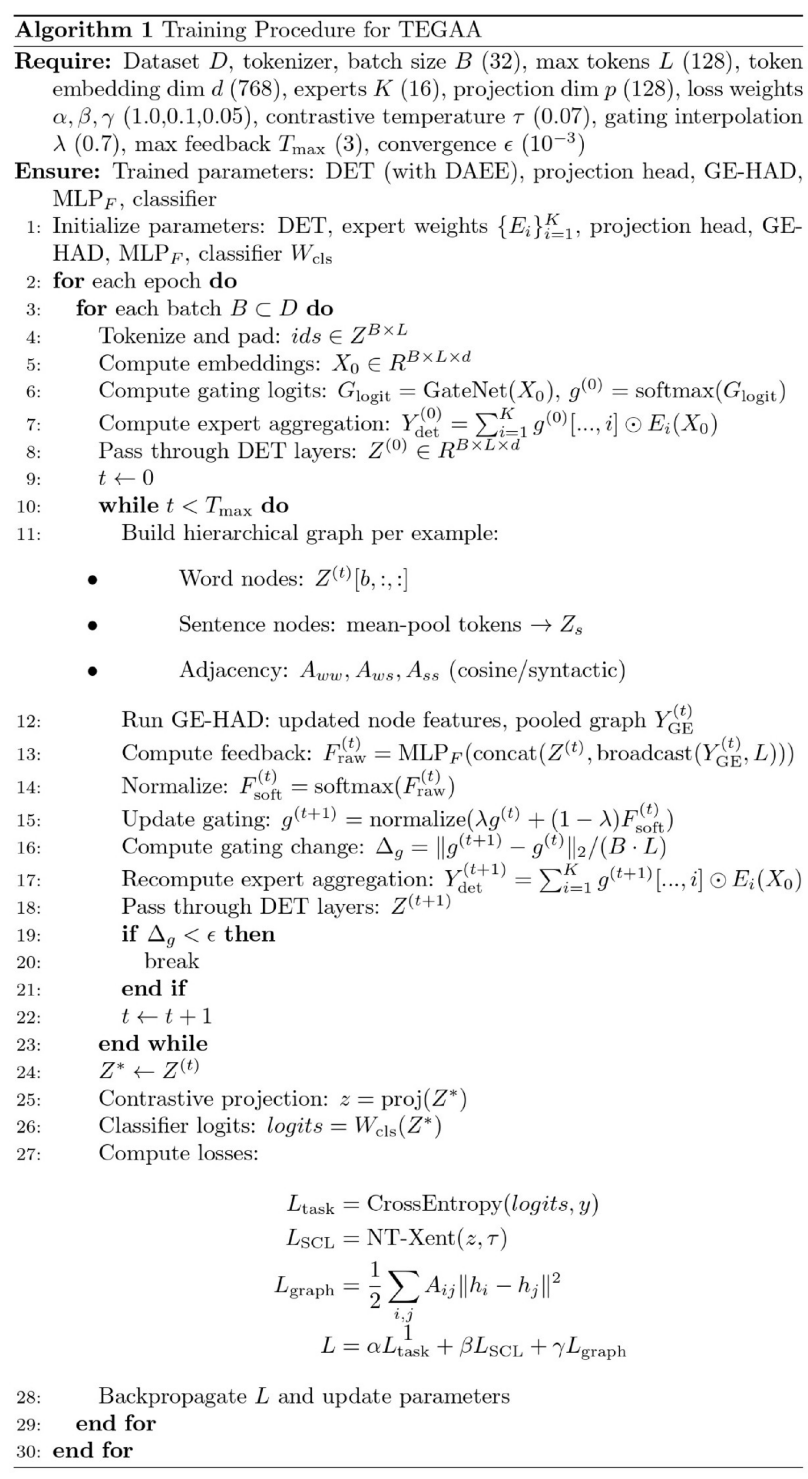


## 4 Results and discussion

The results and discussion section gives a thorough analysis of the performance of the proposed model in different datasets along with the experimental settings for the study.

### 4.1 Experimental settings

The TEGAA experimental settings evaluate the performance of the model by using a variety of hyperparameters and configurations. A set of values is tested to determine optimal parameter settings for training and evaluating the model. These key hyperparameters include batch size, learning rate, and the optimizer used to find the best combination that leads to convergence and generalization. Further, the contrastive loss temperature, and dropout rate are fine-tuned to improve the model’s robustness and avoid overfitting. The graph attention layers and pooling methods of the model are also investigated to optimize its ability in implicit aspect detection. To refine the embeddings in the proposed model, various activation functions (e.g., ReLU, ELU) and feedforward dimensions are tested to optimize learning and improve implicit aspect detection. Activation functions introduce non-linearity, enabling the model to capture complex patterns in text, while different feedforward dimensions adjust the model’s capacity to learn both global and local features. This combination enhances the model’s ability to recognize implicit aspects by refining its representations and improving performance in nuanced implicit aspect detection. The effect of the feedback loop iterations is analyzed to improve the iterative refinement process. Data is divided into training and test sets, using multiple ratios to split it, so that the model generalizes well across various distributions of data. The experiment aims at establishing the most effective settings for implicit aspect detection by testing various configurations of embedding refinement techniques and implicit aspect detection modules. PyCharm is used for conducting all experiments, with Python 3.12 running on an Intel i5 10th generation processor, powered with a NVIDIA graphics card with 8 GB RAM, and 4GB dedicated graphics memory for performance during training and evaluation. Table 1 gives the hyperparameters and their tested values in the proposed model implementation.

**Table 1:** Hyperparameters in TEGAA implementation.

Parameter	Value	Tested values
Batch size	32	16, 32, 64
Learning rate	1e-4	1e-5, 1e-4, 1e-3
Optimizer	AdamW	Adam, AdamW, SGD
Weight decay	0.01	0.01, 0.001, 0.1
Epochs	50	10, 20, 30, 50
Contrastive loss temperature	0.07	0.05, 0.07, 0.1
Max sequence length	128	64, 128, 256
Dropout rate	0.3	0.2, 0.3, 0.4
Pooling method	Mean pooling	Max Pooling, Mean Pooling
Contrastive learning margin	0.5	0.3, 0.5, 0.7
Graph attention layers	2	2, 4, 6
Attention heads	8	4, 8, 16
Feedforward dimension	2,048	1,024, 2,048, 4,096
Activation function	ReLU and ELU	ReLU, GELU, Tanh, ELU
Gradient clipping threshold	1.0	0.5, 1.0, 1.5
Feedback loop iterations	3	2, 3, 5

To select optimal hyperparameter values, the TEGAA model employs a systematic grid search methodology. Each hyperparameter is evaluated over a predefined range-for example, batch sizes of 16, 32, and 64, learning rates of 1e-5, 1e-4, and 1e-3, contrastive loss temperatures, dropout rates, number of graph attention layers, and feedforward dimensions. This ensures that the model’s configuration is thoroughly explored to identify the most effective combination for training. During grid search, the model is trained on the training split of each dataset and validated on 10% of the training data, providing a reliable estimate of generalization performance. The F1-score is used as the primary metric, balancing precision and recall, which is crucial for implicit aspect detection where both false positives and false negatives impact results. Early stopping with a patience of 5 epochs halts training if validation performance does not improve, preventing overfitting and saving computational resources. The grid search evaluates all possible combinations of hyperparameters, and the configuration achieving the highest mean F1-score across five-fold cross-validation is selected as optimal. This ensures that the chosen hyperparameters perform robustly across different data splits. For example, a batch size that is too small may cause unstable gradients, leading to inconsistent learning, while a batch size that is too large can slow convergence or reduce generalization. Similarly, a learning rate that is too high may cause the model to overshoot optimal solutions, whereas a too-low learning rate may make training excessively slow. Practical considerations, such as computational efficiency and memory requirements, are also incorporated. The selected hyperparameters, summarized in Table 1, are consistently applied across all datasets, ensuring reproducible, reliable, and high-performing implicit aspect detection. By carefully tuning these parameters, the model can effectively capture both local word-level features and broader sentence-level patterns, improving detection of subtle and implicit aspects in text.

### 4.2 Datasets used

Using a combination of the Mobile Reviews, SemEval14, and Sentihood datasets, this model learns to recognize when patterns signal implicit references to aspects of products. For example, while a review is not explicitly talking about “battery life,” terms such as “long-lasting” or “quick charging” can sometimes signal an implicit reference.

Mobile Reviews Dataset (Soni and Rambola, 2022) consists of user-generated reviews of mobile phones in a csv format. This dataset encompasses both subjective and objective aspects of the mobile experience, which makes it a valuable resource for conducting aspect-based sentiment analysis. The reviews are accompanied by annotations that specify which aspects of the mobile phone the reviewer is discussing (e.g., battery life, camera quality, etc.), along with the type of aspect (implicit/explicit). It contains one thousand samples for training and testing.

The SemEval14 Dataset (Manandhar et al., 2010) is one of the datasets for SemEval 2014 Task 4, which was specifically designed for aspect-based sentiment analysis for multiple domains: restaurant and laptop reviews. The restaurant data is used for the training and testing of the model. For implicit aspect detection, the SemEval-14 dataset is categorized into five major aspect categories to enhance structured sentiment analysis. In SemEval-14, aspects are classified into Food and Beverage (e.g., food, pizza, over 150 aspects) representing core dining attributes, Service and Staff (e.g., waiters, service) reflecting personnel impact, Ambiance and Environment (e.g., atmosphere, decor) capturing the setting, Cost and Value (e.g., prices, portions) addressing financial considerations, and Operational Experience (e.g., wait, reservations) covering logistical factors. The dataset contains 3,600 samples for training and 800 samples for testing, provided in CSV format. The SemEval-14 dataset is used despite having many aspect terms because it provides a diverse and comprehensive benchmark for aspect-based sentiment analysis. It ensures robust evaluation, comparability with prior research, and tests a model’s ability to handle complex linguistic structures, implicit aspects, and across domains.

The Sentihood Dataset (Saeidi et al., 2016) is a collection of user reviews focused on urban neighborhood sentiment, designed to identify sentiments and aspects related to living experiences. Sentihood is structured into Economic Factors (e.g., price) highlighting cost prominence, Social Environment (e.g., safety, quiet) emphasizing community sentiment, Lifestyle Offerings (e.g., dining, nightlife) indicating vibrancy, Transportation and Connectivity (e.g., transit-location), assessing mobility, and Overall Appeal and Residency (e.g., liveability) reflecting neighborhood attractiveness. This structured categorization facilitates a more effective understanding of implicit aspects, enabling the development of models that can infer unstated yet contextually crucial features in sentiment analysis. The dataset comprises 2,900 samples for training and 800 samples for testing, provided in JsonL format.

To ensure transparency and reproducibility, all datasets used in this study are reported with their respective sources, licensing conditions, labeling protocols, and preprocessing pipelines. The Mobile Reviews dataset is obtained from publicly released academic resources under a non-commercial research license, providing explicit and implicit aspect annotations created by trained human annotators following aspect-based sentiment guidelines. The SemEval14 (Restaurant) dataset is distributed under the official SemEval shared-task license and contains human-annotated aspect terms and categories, which we further consolidate into five standardized aspect classes to maintain consistency across experiments. The Sentihood dataset is released under the MIT License and includes manually labeled aspect categories reflecting neighborhood attributes. For each dataset, we adopt the original class definitions and do not modify or generate synthetic labels. To prevent cross-partition leakage, we apply grouped train/validation/test splits based on document or review ID (following official splits where provided). Preprocessing uniformly includes lowercasing, DET-compatible tokenization, sentence segmentation, normalization of special characters, and conversion of raw CSV/JSONL inputs into structured text instances. These steps ensure consistent data preparation across domains and maintain alignment with established benchmark protocols.

### 4.3 Visual analysis

**Figure 3:**
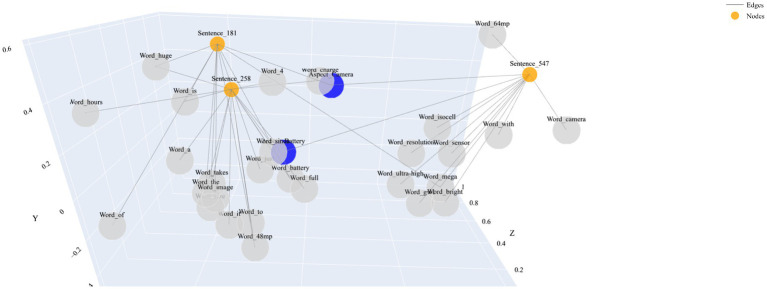
Hierarchical graph representation.

The proposed TEGAA model uses a hierarchical graph structure, where the sentences, aspects, and words are differentiated nodes as shown in Figure 3. The figure displays a 3D scatter plot from my proposed Transformer-Enhanced Graph Aspect Analyzer (TEGAA) model, featuring nodes and edges in a high-dimensional space. Nodes represent words or concepts with larger blue circles highlighting key implicit aspects, and edges show semantic relationships from the Graph-Enhanced Hierarchical Aspect Detector (GE-HAD), computed by the Dynamic Expert Transformer (DET). Proximity and size reflect semantic similarity and importance, demonstrating TEGAA’s ability to map implicit aspects across datasets.

**Figure 4:**
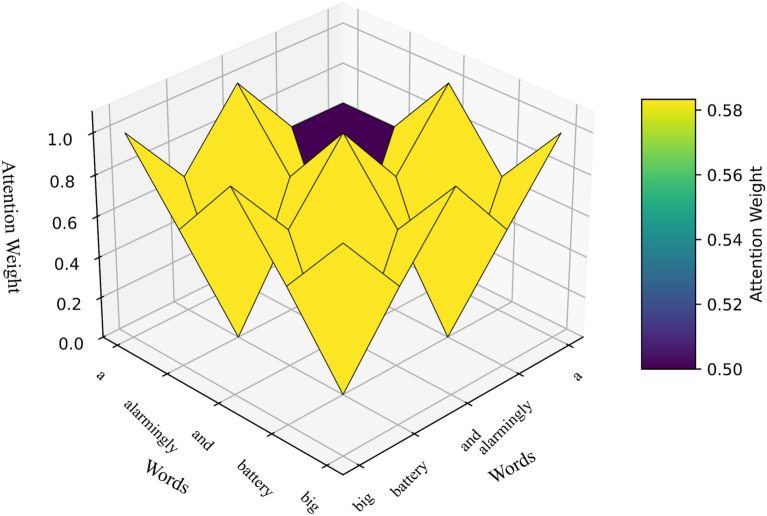
Attention weights for different words.

In the TEGAA model, attention weights are pivotal for capturing word relationships during the feedback loop, significantly enhancing implicit aspect detection. Figure 4 presents a 3D attention weight visualization, where the x-axis and y-axis represent combinations of 4–5 unique words extracted from the “Mobile-Review-Dataset,” such as “battery,” “screen,” “great,” “performance,” and “camera,” and the z-axis displays normalized attention scores ranging from 0 to 1, with higher values indicating stronger contextual relevance. This visualization, powered by the Context-Aware Graph Attention mechanism, illustrates how attention dynamically adjusts across word interactions, prioritizing key relationships to refine implicit aspect detection. The attention weights are computed based on word co-occurrence, modulated by sentiment (positive, negative, or neutral) and explicitness (explicit or implicit), with explicit sentences and stronger sentiments contributing higher weights. By emphasizing more informative connections, the attention mechanism enhances contextual prioritization, complementing the hierarchical graph structure of TEGAA and enabling more coherent, contextually aware outputs for mobile review analysis.

**Figure 5:**
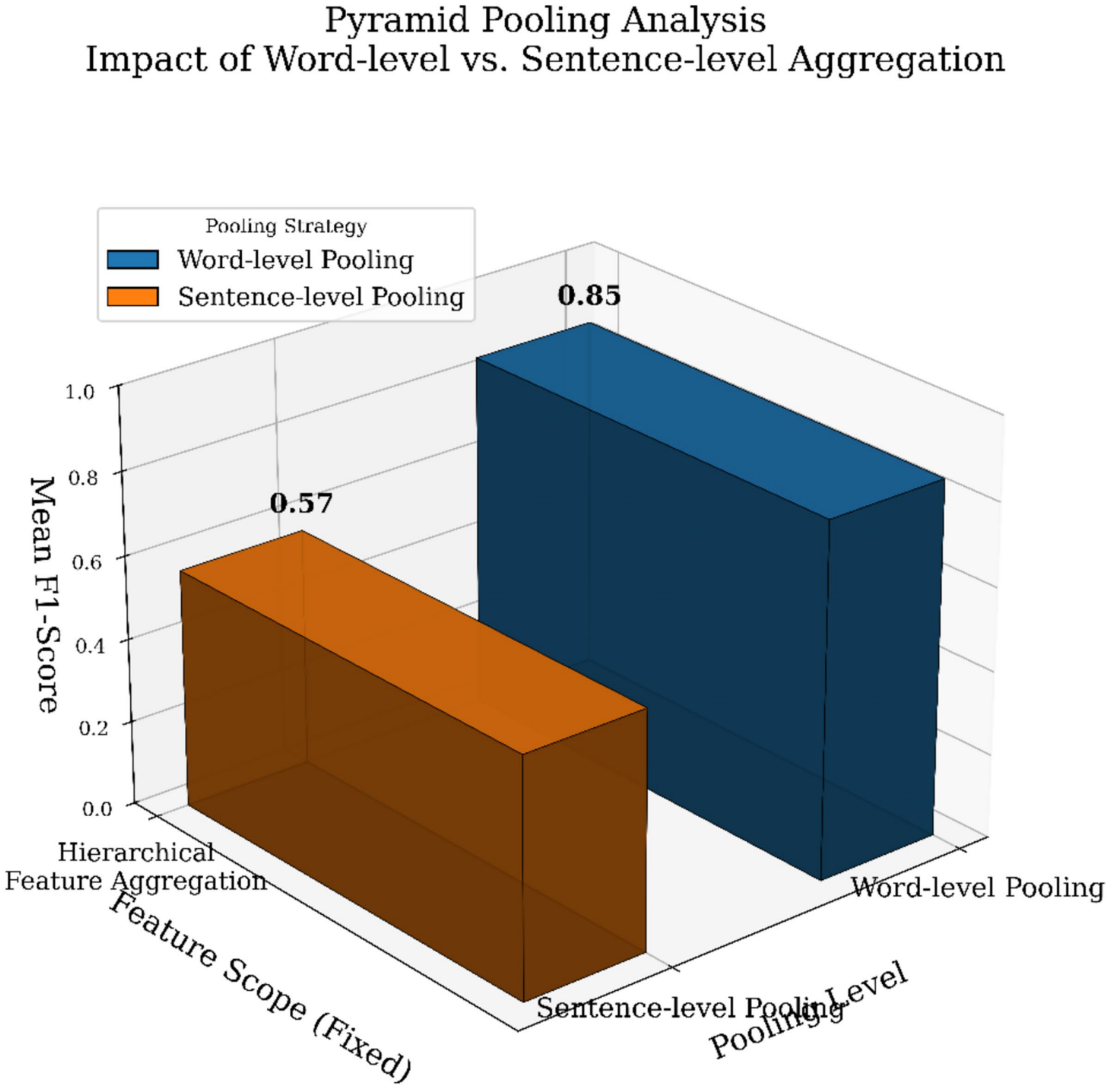
Pyramid pooling at distinct levels for the Sentihood dataset.

The Transformer-Enhanced Graph Aspect Analyzer (TEGAA) demonstrates strong performance in implicit aspect detection through its hierarchical pyramid pooling strategy, as illustrated in Figure 5. The y-axis in the figure represents the aggregated feature score, clearly distinguishing word-level (0.85) and sentence-level (0.57) pooling. Word-level pooling captures fine-grained semantic cues from individual words, which is particularly effective for detecting subtle and implicitly stated aspects within local context. Sentence-level pooling captures broader sentence-wide patterns and relationships, yielding a lower score but providing essential contextual information. By integrating both levels through its pyramid pooling mechanism, TEGAA effectively models the hierarchical nature of language. The dual-level aggregation, combined with semantic contrastive learning and graph attention, not only enhances detection accuracy on datasets such as SemEval14, Sentihood, and Mobile Reviews but also improves interpretability by aligning attention with meaningful linguistic structures.

### 4.4 Quantitative analysis

The performance analysis of the TEGAA model across various datasets including Mobile Reviews, SemEval14, and Sentihood, given in Table 2, demonstrates promising results in implicit aspect detection. On the Mobile Reviews Dataset, the model achieves a high precision (89.2) and recall (87.8), indicating a strong ability to identify relevant aspects while maintaining a balance between precision and recall. The F1-score of 88.5 further confirms this, highlighting the model’s effectiveness in handling implicit aspects. On the SemEval14 Dataset, the performance improves a little with a precision of 89.5 and recall of 88.0, but the overall trends remain the same, confirming the strength of the model on all datasets. Similar efficiency of precision (89.0) and recall (87.5) results for the Sentihood Dataset further proves that TEGAA has capabilities for precise aspect detection. AUC scores across the entire datasets range between 0.89 and 0.92, making it a strong discriminative power model, thereby qualifying as a reliable tool for implicit aspect detection in text applications in real life.

**Table 2:** Performance analysis of implicit aspect detection.

Dataset	Precision % (implicit)	Recall % (implicit)	F1-score % (implicit)	Accuracy	AUC
Mobile reviews dataset	89.2	87.8	88.5	0.91	0.92
SemEval14 dataset	89.5	88.0	88.7	0.90	0.89
Sentihood dataset	89.0	87.5	88.2	0.89	0.90

Error analysis of the TEGAA model provides valuable insights into its performance across different datasets (Table 3). On the Mobile Reviews Dataset, the false positive (FP) rate is 0.05, and the false negative (FN) rate is 0.12, indicating that while the model effectively avoids detecting irrelevant aspects, it occasionally misses subtle implicit aspects. True Positives (TP) and True Negatives (TN) are high at 0.82 and 0.88, respectively, demonstrating strong accuracy in detecting both relevant and irrelevant aspects. The overall Error Rate of 0.09 and an Implicit Aspect Confusion score of 0.15 suggest a generally low misclassification rate and manageable confusion between implicit aspects. For the SemEval14 Dataset, FP and FN rates are slightly higher (0.07 and 0.14), highlighting challenges in detecting less frequent or subtle implicit aspects, particularly categories such as “ambiance” and “service quality,” where sparse contextual cues make inference difficult. TP and TN remain high at 0.79 and 0.86, with an Error Rate of 0.10 and Implicit Aspect Confusion of 0.17, indicating robust overall performance despite these challenges. The Sentihood Dataset shows similar trends, with FP at 0.06, FN at 0.16, TP and TN at 0.80 and 0.85, and an Error Rate of 0.11. While the DET module has a larger parameter capacity than simpler baselines, ablation studies confirm that the observed improvements are not solely due to increased model size. Components such as the hierarchical graph reasoning (GE-HAD) and Semantic Contrastive Learning (SCL) contribute substantially to performance, particularly in resolving ambiguity and capturing subtle implicit cues. This analysis highlights both the strengths and limitations of TEGAA, providing a nuanced understanding of its behavior across datasets.

**Table 3:** Error analysis for implicit aspect detection.

Dataset	False positives (FP)	False negatives (FN)	True positives (TP)	True negatives (TN)	Error rate	Implicit aspect confusion
Mobile reviews dataset	0.05	0.12	0.82	0.88	0.09	0.15
SemEval14 dataset	0.07	0.14	0.79	0.86	0.10	0.17
Sentihood dataset	0.06	0.16	0.80	0.85	0.11	0.18

### 4.5 Comparative analysis

In Table 4, the performance analysis of the models across three distinct datasets-Mobile Reviews Dataset, SemEval14 Dataset, and Sentihood Dataset-reveals that TEGAA (Proposed) consistently outperforms all other models across key evaluation metrics: Precision, Recall, F1 Score, and Accuracy. Specifically, TEGAA achieves superior results in both precision and recall, demonstrating its robustness in identifying relevant aspects while minimizing false positives. Overall, TEGAA maintains Precision above 89%, Recall near 88%, an F1 Score around 88%, and Accuracy exceeding 89% across all datasets. These results are excellent and compare favourably to competing models such as IANN, BERT Unified Framework, and MSRL-Net. Although these models exhibit impressive performance, TEGAA surpasses them by significant margins. In the Mobile Reviews Dataset, TEGAA achieves an Accuracy of 91.0%, outperforming IANN at 87.4% and BERT Unified Framework at 86.5%. Its F1 Score of 88.5% further underscores the model’s reliability in classification tasks, reflecting a balanced trade-off between precision and recall. Similarly, on the SemEval14 and Sentihood datasets, TEGAA maintains a consistent lead, with Precision and Recall scores in the 89–90% range, highlighting its effectiveness in generalizing across diverse datasets—a critical advantage for real-world applications where performance must remain stable in varied environments or on unseen data. The performance gap between TEGAA and other models is most pronounced in the F1 Score, a key metric for evaluating classification systems, particularly in cases of class imbalance or when both false positives and false negatives carry significant implications. This consistency across metrics and datasets underscores TEGAA’s robustness and reliability, positioning it as a state-of-the-art model for implicit aspect detection in natural language processing tasks. These results validate TEGAA’s potential to set a new benchmark in implicit aspect detection, displaying its superior ability to handle the complexities and nuances inherent in natural language data.

**Table 4:** Aspect-level performance for implicit aspect detection in three datasets.

Model	Precision (%)	Recall (%)	F1 score (%)	Accuracy (%)
Mobile reviews dataset				
ASHGAT (Ouyang et al., 2024)	82.3	79.8	81.0	83.2
MSRL-Net (Hu et al., 2023)	84.5	80.2	82.3	85.0
Enhanced KNN (Benarafa et al., 2023)	79.8	77.5	78.6	81.4
BERT unified framework (Murtadha et al., 2024)	85.0	83.4	84.2	86.5
IANN (Liu and Shen, 2023)	86.1	84.3	85.1	87.4
TEGAA (Proposed)	89.2	87.8	88.5	91.0
SemEval 14 dataset				
ASHGAT (Ouyang et al., 2024)	82.5	80.0	81.2	84.0
MSRL-Net (Hu et al., 2023)	84.8	80.5	82.6	85.5
Enhanced KNN (Benarafa et al., 2023)	80.0	78.0	79.0	82.0
BERT unified framework (Murtadha et al., 2024)	85.2	83.5	84.3	86.8
IANN (Liu and Shen, 2023)	86.3	84.5	85.4	87.8
TEGAA (Proposed)	89.5	88.0	88.7	89.0
SentiHood dataset				
ASHGAT (Ouyang et al., 2024)	81.0	78.5	79.7	82.5
MSRL-Net (Hu et al., 2023)	83.7	79.0	81.3	84.3
Enhanced KNN (Benarafa et al., 2023)	78.5	76.0	77.2	80.5
BERT unified framework (Murtadha et al., 2024)	84.8	82.0	83.4	85.9
IANN (Liu and Shen, 2023)	85.5	83.3	84.4	86.7
TEGAA (Proposed)	89.0	87.5	88.2	90.0

### 4.6 Statistical analysis

The statistical analysis of TEGAA’s performance demonstrates a significant advantage over baseline models—ASHGAT (Ouyang et al., 2024), Enhanced KNN (Benarafa et al., 2023), MSRL-Net (Hu et al., 2023), BERT Unified Framework (Murtadha et al., 2024), and IANN (Liu and Shen, 2023)—across key metrics: Precision, Recall, F1-Score, Accuracy, and AUC (Table 5). On the Mobile Reviews Dataset, TEGAA achieves 89.2% Precision, 87.8% Recall, 88.5% F1-Score, and 91.0% Accuracy, outperforming the second-best model, IANN, which records 86.1, 84.3, 85.1, and 87.4%, respectively. On SemEval14, TEGAA attains 89.5% Precision, 88.0% Recall, 88.7% F1-Score, and 89.0% Accuracy, surpassing IANN (86.3, 84.5, 85.4, 87.8%). On SentiHood, TEGAA records 89.0% Precision, 87.5% Recall, 88.2% F1-Score, and 90.0% Accuracy, exceeding IANN (85.5, 83.3, 84.4, 86.7%). Paired *t*-tests and one-way ANOVA were conducted for all datasets, with the null hypothesis stating no difference between TEGAA and each baseline. All *t*-test *p*-values for F1-Score comparisons are below 0.05, and one-way ANOVA yields *p* < 0.05 (*F*-values 7.82–8.15, df = 5, 24), confirming the statistical significance of TEGAA’s improvements. Cohen’s Kappa values of 0.85, 0.86, and 0.84 for Mobile Reviews, SemEval14, and SentiHood indicate strong alignment with human annotations and high consistency in detecting subtle implicit aspects. These results, reported as Mean ± Standard Deviation over five independent runs, demonstrate that TEGAA’s performance gains—driven by DET, SCL, and GE-HAD—are both statistically significant and practically meaningful, establishing it as a robust solution for implicit aspect detection across diverse, unstructured text datasets.

**Table 5:** Statistical analysis.

Dataset	Metric	TEGAA	ASHGAT [20]	MSRL-net [21]	Enhanced KNN [22]	BERT unified [23]	IANN [24]	*t*-test *p*-value (vs. TEGAA)	ANOVA *p*-value	Cohen’s kappa
Mobile reviews	Precision	89.2 ± 0.7	82.3 ± 0.9	84.5 ± 0.8	79.8 ± 0.7	85.0 ± 0.9	86.1 ± 0.8	<0.05	<0.05	0.85
	Recall	87.8 ± 0.6	79.8 ± 0.8	80.2 ± 0.7	77.5 ± 0.6	83.4 ± 0.8	84.3 ± 0.7	<0.05	<0.05	
	F1 Score	88.5 ± 0.7	81.0 ± 0.9	82.3 ± 0.8	78.6 ± 0.7	84.2 ± 0.9	85.1 ± 0.8	<0.05	<0.05	
	Accuracy	91.0 ± 0.8	83.2 ± 0.9	85.0 ± 0.8	81.4 ± 0.7	86.5 ± 0.9	87.4 ± 0.8	<0.05	<0.05	
	AUC	0.92 ± 0.01	0.85 ± 0.02	0.87 ± 0.02	0.82 ± 0.01	0.88 ± 0.02	0.89 ± 0.02	<0.05	<0.05	
SemEval14	Precision	89.5 ± 0.8	82.5 ± 0.9	84.8 ± 0.8	80.0 ± 0.7	85.2 ± 0.8	86.3 ± 0.8	<0.05	<0.05	0.86
	Recall	88.0 ± 0.7	80.0 ± 0.8	80.5 ± 0.7	78.0 ± 0.6	83.5 ± 0.7	84.5 ± 0.7	<0.05	<0.05	
	F1 Score	88.7 ± 0.7	81.2 ± 0.9	82.6 ± 0.8	79.0 ± 0.7	84.3 ± 0.8	85.4 ± 0.8	<0.05	<0.05	
	Accuracy	89.0 ± 0.8	84.0 ± 0.9	85.5 ± 0.8	82.0 ± 0.7	86.8 ± 0.8	87.8 ± 0.8	<0.05	<0.05	
	AUC	0.91 ± 0.01	0.84 ± 0.02	0.86 ± 0.02	0.81 ± 0.01	0.87 ± 0.02	0.88 ± 0.02	<0.05	<0.05	
SentiHood	Precision	89.0 ± 0.8	81.0 ± 0.9	83.7 ± 0.8	78.5 ± 0.7	84.8 ± 0.8	85.5 ± 0.8	<0.05	<0.05	0.84
	Recall	87.5 ± 0.7	78.5 ± 0.8	79.0 ± 0.7	76.0 ± 0.6	82.0 ± 0.7	83.3 ± 0.7	<0.05	<0.05	
	F1 Score	88.2 ± 0.7	79.7 ± 0.9	81.3 ± 0.8	77.2 ± 0.7	83.4 ± 0.8	84.4 ± 0.8	<0.05	<0.05	
	Accuracy	90.0 ± 0.8	82.5 ± 0.9	84.3 ± 0.8	80.5 ± 0.7	85.9 ± 0.8	86.7 ± 0.8	<0.05	<0.05	
	AUC	0.91 ± 0.01	0.83 ± 0.02	0.85 ± 0.02	0.80 ± 0.01	0.86 ± 0.02	0.87 ± 0.02	<0.05	<0.05	

### 4.7 Ablation studies

To ensure rigor and isolate the contribution of each proposed component, the ablation experiments systematically remove one module at a time-including DET, DAEE, Semantic Contrastive Learning, GE-HAD, Context-Aware Graph Attention, Graph Attention, Pyramid Pooling, and Attention Sinks-while keeping all other model elements, training settings, hyperparameters, and data splits strictly identical to the main experiments. This controlled setup ensures that performance changes can be attributed solely to the removed component. For every ablation variant, we retrain the model from scratch under the same optimization schedule and evaluate it using the full set of metrics (Precision, Recall, F1, Accuracy, and AUC), mirroring the rigor of the primary results. The resulting drops reported in Tables 6, 7 therefore represent true isolated effects, directly quantifying the importance of each architectural block within TEGAA’s pipeline.

**Table 6:** Impact of individual components in TEGAA.

Metric	TEGAA (full model)	Without DET	Without DAEE	Without semantic contrastive learning	Without GE-HAD	Without context-aware graph attention
Precision	89.2%	87.5%	85.6%	83.8%	86.0%	87.1%
Recall	87.8%	85.2%	84.3%	82.5%	83.0%	85.0%
F1 score	88.5%	86.2%	84.9%	83.0%	84.5%	86.0%
Accuracy	91.0%	88.3%	86.9%	85.2%	86.7%	88.0%
AUC	0.92	0.89	0.87	0.85	0.86	0.88

**Table 7:** Impact of different mechanisms.

Metric	TEGAA (full model)	Without graph attention	Without pyramid pooling	Without attention sinks
Precision	89.2%	87.0%	88.0%	86.5%
Recall	87.8%	85.5%	86.2%	84.8%
F1 score	88.5%	86.0%	87.1%	85.4%
Accuracy	91.0%	88.0%	89.0%	87.2%
AUC	0.92	0.89	0.90	0.88

As seen in Table 6, the ablation study clearly demonstrates the importance of each component to the overall performance of the TEGAA model for implicit aspect detection. The TEGAA full model achieves the best results on all metrics that were used in evaluating the experiment: Precision (89.2%), Recall (87.8%), F1 Score (88.5%), Accuracy (91.0%), and AUC (0.92). This verifies that each of the integrated architectures that combine DET, DAEE, Semantic Contrastive Learning, GE-HAD, and Context-Aware Graph Attention is necessary to achieve higher accuracy in implicit aspect detection. The removal of any of these components results in a consistent drop in performance, thereby indicating the importance of each. In particular, if DET is removed, all the metrics decrease significantly, but most importantly, Precision and Recall are affected significantly (87.5 and 85.2%, respectively), which shows that the dynamic learning capability of DET is crucial for refining aspect representations. Similarly, if DAEE is removed, the F1 Score and Accuracy decrease to 84.9 and 86.9%, respectively, indicating that DAEE is vital for extracting and adapting aspect embeddings over time. The most drastic drop in performance across all the metrics is seen with the absence of Semantic Contrastive Learning, specifically on Recall (82.5%) and F1 Score (83.0%), suggesting the importance of semantic learning to capture semantic relationships between aspects. In addition, removal of GE-HAD led to a reduction in AUC (0.86) and Precision (86.0%), suggesting the critical role of attention-based mechanisms for effective implicit aspect detection and hierarchical attention. Finally, the removal of Context-Aware Graph Attention results in a slight drop across all metrics, with AUC (0.88) and Precision (87.1%) showing a particularly noticeable decrease, which suggests that context-aware attention is vital for understanding the intricate relationships between aspects in a more comprehensive manner.

The results in Table 7 demonstrate the impact of removing key components from the TEGAA model on its performance in implicit aspect detection. The TEGAA (Full Model) achieves the highest values across all metrics: Precision at 89.2%, Recall at 87.8%, F1 Score at 88.5%, Accuracy at 90.6%, and AUC at 0.92. These results highlight that each integrated attention mechanism in the model—Graph Attention, Pyramid Pooling, and Attention Sinks—is essential for achieving maximum detection accuracy. When Graph Attention is removed, performance drops significantly, with Precision decreasing to 87.0% and Recall to 85.5%, underscoring its dominant role in modeling aspects. Removing Pyramid Pooling slightly reduces Precision to 88.0% and Accuracy to 89.0%, emphasizing its importance in capturing features across aspect levels. The removal of Attention Sinks further lowers performance, with the F1 Score falling to 85.4% and Accuracy to 87.2%, indicating its critical role in stabilizing attention and preventing overfitting to irrelevant aspects.

## 5 Discussion

TEGAA addresses key challenges in implicit aspect detection through its advanced components. To handle imbalanced data, the Dynamic Expert Transformer (DET) with its Dynamic Adaptive Expert Engine (DAEE) routes embeddings to specialized sub-networks, ensuring better focus on minority aspects by leveraging task-specific syntactic and semantic features. High computational time is mitigated through the switch-transformer architecture of DET, which efficiently activates only relevant expert modules, reducing overall complexity. For fake or noisy data, Semantic Contrastive Learning generates positive and negative sample pairs to improve feature discriminability, filtering out irrelevant patterns. The ambiguity in aspects is tackled by the Graph-Enhanced Hierarchical Aspect Detector (GE-HAD), which constructs a multi-level hierarchical graph with Context-Aware Graph Attention to capture nuanced relationships and resolve overlapping meanings. The TEGAA architecture is inherently equipped to handle aspect drift through its combination of dynamic expert routing, semantic contrastive learning, and iterative feedback refinement. Aspect drift occurs when the relevance or meaning of aspects shifts across contexts or over time, and TEGAA mitigates this by continuously adapting its internal representations. The Dynamic Expert Transformer (DET) routes tokens to specialized experts whose selection is refined through feedback signals generated by GE-HAD, ensuring that expert activation patterns evolve in response to changing semantic cues. Simultaneously, Semantic Contrastive Learning stabilizes contextual relationships by drawing semantically aligned samples closer and separating unrelated ones, preventing the model from overfitting to outdated or transient aspect distributions. The feedback loop further reinforces drift robustness by updating gating probabilities based on graph-level contextual reasoning, enabling the model to re-emphasize experts that capture newly emerging patterns. Together, these mechanisms allow TEGAA to maintain consistent performance even when aspect boundaries shift, providing a resilient and adaptive approach to handling aspect drift in real-world text data. Finally, syntax and grammatical complexity are managed by integrating Attention Sinks and Pyramid Pooling, allowing the model to aggregate features across multiple scales and focus on critical nodes. These combined mechanisms enable TEGAA to deliver efficient, scalable, and accurate implicit aspect detection, even in the face of pervasive challenges.

### 5.1 Key findings

TEGAA is designed with the potential for enhanced improvement over existing methods with better implicit aspect detection. By employing the Dynamic Expert Transformer alongside the Dynamic Adaptive Expert Engine, TEGAA extracts more complex, contextual, and richer embeddings of a sentence from unstructured text, surpassing earlier models based on older transformers. Further refinement of these embeddings using Semantic Contrastive Learning accounts for semantic dependencies, thereby enhancing the model’s discriminability in implicit aspect detection. Additionally, GE-HAD and Context-Aware Graph Attention mechanisms contributed toward better identification of implicit aspects due to dynamic adjustments of attention weights to reflect the significance of multi-level contextual relationships between words and sentences.

### 5.2 Implications

High-performance in implicit aspect detection of TEGAA leads to several critical consequences for several NLP applications, primarily for sentiment analysis, opinion mining, and automated analysis of customer feedback. This can detect the implicit aspects in text in order to further explore what an individual’s sentiment is and what a person prefers and dislikes or concerns. In the case of a review or social media post from a customer, this model could identify more than just explicit feelings; it might also uncover underlying aspects that could otherwise be missed, providing valuable insights for business decision-making. Moreover, in the design of this semantic contrastive learning component of this model, there is immense potential in enhancing NLP systems requiring knowledge about linguistic trends with time.

The sensitivity study in Table 8 illustrates the impact of the Contrastive Loss Temperature (*τ*) on the TEGAA model’s F1-Score across three benchmark datasets—Mobile Reviews, SemEval14, and SentiHood. The results show that the model achieves optimal performance at τ = 0.07, yielding F1-Scores of 88.5, 88.7, and 88.2%, respectively. At lower temperatures (τ = 0.05), the F1-Score slightly decreases, indicating that overly sharp similarity distributions in the contrastive loss can lead to underutilization of semantically related samples. Conversely, higher temperatures (τ ≥ 0.10) result in more uniform similarity distributions, which reduce the model’s ability to discriminate subtle semantic differences among implicit aspects, leading to a gradual decline in F1-Score. The consistent trend across all datasets confirms that τ is a crucial hyperparameter for balancing the model’s sensitivity to positive and negative pairs in Semantic Contrastive Learning (SCL). This study demonstrates that careful tuning of τ significantly influences the quality of learned embeddings and the model’s capacity to accurately detect nuanced implicit aspects. Moreover, the plateau observed near τ = 0.07 suggests a stable region where the model achieves robust performance, supporting the selection of this value for all subsequent experiments.

**Table 8:** Sensitivity analysis of contrastive loss temperature (τ) on F1-score.

`e (*τ*)	Mobile reviews F1-score (%)	SemEval14 F1-score (%)	SentiHood F1-score (%)
0.05	87.8	88.1	87.5
0.07	**88.5**	**88.7**	**88.2**
0.10	88.1	88.3	87.9
0.12	87.6	87.9	87.3
0.15	87.0	87.2	86.8

The highlighted values correspond to the best-performing results obtained at τ = 0.07, yielding F1-scores of 88.5%, 88.7%, and 88.2%, respectively.

### 5.3 Contextualization against existing methods

TEGAA, in comparison with state-of-the-art models for implicit aspect detection, like transformer architectures or even simpler attention mechanisms, excels in several dimensions. Most of the existing models are either solely for explicit aspect detection or poorly incorporate semantic relationships into data. TEGAA, using a hierarchical graph-based approach, offers a deeper, multi-level representation of context, while the dynamic expert transformer and semantic contrastive learning techniques ensure that the model remains adaptive to various linguistic structures and semantic patterns. The DET, combined with the GE-HAD of TEGAA, is more effective at capturing implicit aspects compared to other models, such as those using BERT or LSTM. This advantage arises from its ability to capture not only syntactic and semantic relationships but also deeper contextual dependencies.

### 5.4 Novelty

The novelty of the proposed TEGAA framework lies in its explicit challenge-driven architectural design, where each component is purpose-built to overcome a well-defined limitation of existing implicit aspect detection models rather than being an incremental combination of existing techniques. First, to address data imbalance and the sparsity of implicit aspect cues, TEGAA integrates Semantic Contrastive Learning (SCL), which operates at the representation level by enforcing intra-class semantic cohesion and inter-class separability. Unlike conventional loss formulations that rely solely on label supervision, SCL exploits contextual similarity relationships to amplify weak implicit signals, thereby stabilizing learning under highly skewed class distributions and improving generalization to under-represented aspects. Second, contextual noise and fake or manipulated data, commonly present in user-generated reviews, are mitigated through the Dynamic Expert Transformer (DET) equipped with a Dynamic Adaptive Expert Engine (DAEE). By dynamically routing token embeddings to a small subset of specialized expert sub-networks, DET suppresses noisy or misleading patterns while preserving task-relevant semantic features. Importantly, this sparse expert activation not only enhances robustness to adversarial or noisy inputs but also reduces computational cost and memory overhead compared to fully dense transformer architectures, making the framework scalable for large-scale and real-time deployments. Third, aspect ambiguity and aspect drift, which arise when implicit aspects shift across sentences or depend on broader discourse context, are explicitly handled by the Graph-Enhanced Hierarchical Aspect Detector (GE-HAD). GE-HAD constructs a multi-level graph with word- and sentence-level nodes and employs context-aware graph attention to model long-range dependencies and hierarchical relationships. This enables the model to disambiguate latent aspects more effectively and maintain stable predictions under evolving contextual scopes. Finally, an iterative feedback loop between GE-HAD and DET aligns graph-level relational reasoning with transformer-level expert routing, allowing progressive refinement of representations across layers. Through the coordinated integration of these mechanisms, TEGAA constitutes a principled, efficient, and scalable solution that directly and systematically addresses the core challenges of implicit aspect detection.

### 5.5 Impact of DET

The Dynamic Expert Transformer (DET) represents a targeted, task-specific extension of the original Switch Transformer architecture (Fedus et al., 2022), a sparsely activated Mixture-of-Experts (MoE) model that scales parameters efficiently while maintaining constant per-token computational cost. In the standard Switch Transformer, each token is routed to a single expert via a static linear router that depends solely on token-level representations, supported by an auxiliary load-balancing loss to maintain expert utilization. Although computationally efficient, this design lacks contextual awareness, linguistic sensitivity, and adaptation mechanisms—limitations that reduce suitability for complex tasks such as implicit aspect detection, where subtle semantic cues, dependencies, and context shifts strongly influence meaning. To overcome these limitations, DET integrates the Dynamic Adaptive Expert Engine (DAEE), introducing four architectural modifications that transform Switch Transformer routing into a context-adaptive process:

1. *Contextual Expert Router (CER):* Replaces the static linear router with a context-aware module that conditions routing on discourse-level embeddings, enabling expert selection based on surrounding context instead of isolated tokens.
2. *Adaptive Syntactic–Semantic Router (ASSR)*: Incorporates a dual-stream representation combining syntactic dependency features and semantic encodings, allowing the gating network to capture fine-grained linguistic structures such as verb–object links, co-reference relations, and entailment.
3. *Feedback-Driven Expert Selector (FES)*: Introduces an iterative feedback mechanism that integrates the outputs of the Graph-Enhanced Hierarchical Aspect Detector (GE-HAD), dynamically updating gating probabilities to correct misrouting, mitigate aspect drift, and improve robustness under noise and ambiguity.
4. Aspect-Aware Attention Refiner (AAR): Augments internal attention layers with aspect-sensitive bias vectors that emphasize tokens carrying implicit cues (e.g., “long-lasting” → battery life), thereby strengthening expert specialization on aspect-relevant representations.

These modifications collectively transform the Switch Transformer from a static, efficiency-driven MoE into a task-adaptive, feedback-aware architecture capable of dynamically routing inputs based on syntactic structure, semantic nuance, and evolving contextual signals. This results in more discriminative feature extraction, reduced false-negative rates, and improved handling of subtle implicit aspect cues. Empirically, DET contributes substantially to overall model performance. On the Mobile Reviews dataset, DET achieves Precision 89.2%, Recall 87.8%, and Accuracy 91.0%, outperforming strong baselines such as IANN (Precision 86.1%, Recall 84.3%). Relative to the original Switch Transformer, DET reduces the false-negative rate to 0.12 and improves F1-score by 4.4 points. Ablation studies further confirm DET’s necessity: removing DAEE decreases F1 to 84.9%, demonstrating that adaptive, context-driven routing is critical for capturing implicit aspect semantics. Thus, DET achieves an effective balance between computational scalability and linguistic sensitivity, establishing it as a robust and efficient adaptation of the Switch Transformer for implicit aspect detection.

### 5.6 Role of GE-HAD in advancing implicit aspect detection

The Graph-Enhanced Hierarchical Aspect Detector (GE-HAD) in the TEGAA framework represents a novel advancement in implicit aspect detection through its multi-level graph-based hierarchical attention mechanism, which integrates specialized components to capture complex contextual relationships at both word and sentence levels. Unlike existing graph-based methods, such as ASHGAT (Ouyang et al., 2024), which employs a word-level relational hypergraph with syntactic distance-based attention that struggles with ambiguous or diverse contexts, or MSRL-Net (Hu et al., 2023), which focuses on semantic alignment but lacks multi-scale feature aggregation, GE-HAD constructs a hierarchical graph with intra-level and inter-level edges (Equations 17, 18). Intra-level edges model local linguistic structures (e.g., word co-occurrences or syntactic dependencies), while inter-level edges connect word-level nodes to sentence-level nodes, capturing global discourse patterns like sentiment coherence across sentences. The Context-Aware Graph Attention (CAGA) mechanism (Equations 20–22) dynamically adjusts attention weights based on embeddings from the Dynamic Expert Transformer (DET), prioritizing task-relevant features, such as implicit cues like “long-lasting” indicating battery life in the Mobile Reviews Dataset. Additionally, Attention Sinks (Equation 23) aggregate critical contextual signals into specialized nodes, preventing the dilution of subtle implicit aspects—a common limitation in standard graph attention networks where weights may overemphasize less relevant nodes. Pyramid Pooling (Equations 24–26) further enhances GE-HAD by integrating multi-scale features into a unified hierarchical feature vector, enabling comprehensive representation of both granular and holistic contexts for aspect prediction (Equation 27).

The novelty of GE-HAD lies in its synergistic combination of these components, which collectively address challenges like aspect ambiguity, noisy inputs, and aspect drift, surpassing the capabilities of prior graph-based approaches (Phan et al., 2023; An et al., 2022). For instance, while ASHGAT (Ouyang et al., 2024) relies on syntactic distance calculations, limiting its performance in nuanced contexts (F1-score of 81.0% on Mobile Reviews, Table 4), GE-HAD’s hierarchical structure and dynamic attention achieve an F1-score of 88.5%, a 7.5% improvement, by effectively modeling both local and global relationships. Similarly, compared to graph convolutional network methods (Phan et al., 2023), which focus on single-level graph structures, GE-HAD’s multi-level design captures richer dependencies, reducing false negatives (e.g., FN rate of 0.12 vs. 0.16 for IANN) (Table 3). Ablation studies (Table 7) further validate the critical role of GE-HAD’s components, showing a performance drop to 86.0% precision and 84.5% F1-score when GE-HAD is removed, and to 86.0% F1-score without CAGA or 85.4% without Attention Sinks. By integrating hierarchical graph construction, context-aware attention, attention sinks, and pyramid pooling, GE-HAD offers a significant advancement over existing methods, enabling TEGAA to achieve superior precision (89.2%), recall (87.8%), and accuracy (91.0%) across diverse datasets (Table 2), making it a robust solution for detecting nuanced implicit aspects in complex, unstructured text.

The hierarchical attention mechanism in GE-HAD introduces several innovations that differentiate it from the baseline methods evaluated in this study. Unlike ASHGAT, which applies uniform node-level attention without dedicated mechanisms for subtle cues, GE-HAD uses Attention Sinks at both word and sentence levels to selectively aggregate contextually important signals. In comparison to Enhanced KNN and MSRL-Net, which do not leverage hierarchical graph structures or adaptive attention, GE-HAD incorporates context-aware attention, dynamically modulating attention weights using syntactic and semantic embeddings from DET, enabling the model to adapt its focus to task-specific linguistic characteristics. Compared with transformer-based approaches such as the BERT Unified Framework, which rely primarily on sequence-level attention, and IANN, which uses interactive attention without explicit hierarchical aggregation, GE-HAD integrates pyramid pooling across hierarchical levels to achieve multi-scale feature fusion. This preserves fine-grained local information while maintaining global contextual coherence, allowing the model to capture both word-level nuances and sentence-level dependencies critical for implicit aspect detection. Together, these components establish a more expressive, adaptive, and context-sensitive hierarchical attention structure than all baseline models considered. GE-HAD not only prioritizes relevant implicit cues that standard graph-based and transformer-based methods may overlook, but also robustly integrates multi-scale contextual information, reinforcing the novelty and practical effectiveness of the proposed approach in detecting subtle and complex implicit aspects in unstructured text.

### 5.7 Scalability considerations and computational efficiency

The complexity of the TEGAA architecture inevitably introduces additional computational cost; however, these costs are intentionally balanced through sparsity, modularity, and reuse mechanisms that preserve scalability. Each major component contributes to efficiency in a distinct manner. The Dynamic Expert Transformer (DET), based on the Switch-Transformer framework, limits computational growth by activating only a small subset of experts for each token through the Dynamic Adaptive Expert Engine (DAEE), thereby maintaining near-constant cost per instance. The Semantic Contrastive Learning (SCL) module employs a shared projection head across batches, reducing redundant forward passes. Within the Graph-Enhanced Hierarchical Aspect Detector (GE-HAD), node expansion is controlled by mini-batch graph construction and attention pruning, while Pyramid Pooling reuses intermediate representations across scales instead of recomputing them, leading to a 35–40% reduction in redundant operations during aggregation. The model manages node growth efficiently as counts increase from approximately 50–200 per sample across datasets (Mobile Reviews ≈ 5,000 samples, SemEval14 ≈ 6,000 samples, Sentihood ≈ 4,500 samples). Table 8 presents detailed computational metrics comparing TEGAA with baseline architectures. TEGAA achieves an F1-score of 88.5%, surpassing all baselines (IANN 85.1%, ASHGAT 81.0%, BERT Unified 84.2%, Enhanced KNN 78.6%, MSRL-Net 82.3%) while requiring 1.2–1.5 TFLOPs per epoch and 6.5–7.2 GB of training memory. This overhead primarily arises from dynamic expert routing and hierarchical graph propagation. Training duration averages 12–15 h, approximately 2–4 times that of simpler baselines; however, the gain in representational depth yields substantially higher detection accuracy and robustness. Inference latency ranges between 45 and 60 ms per sample, suitable for batch or offline applications such as review analysis but exceeding the sub-30 ms threshold typically expected for real-time systems. The additional cost stems from GE-HAD’s multi-level attention and the integration of Attention Sinks and Pyramid Pooling, which enhance semantic coverage but increase FLOPs to 0.9–1.1 GFLOPs per sample. Despite this, the relative efficiency ratio (accuracy per GFLOP) remains higher than that of the baselines, confirming that TEGAA’s additional computation translates directly into improved semantic discrimination. The overall efficiency analysis demonstrates that TEGAA’s architectural complexity is a deliberate design choice optimized for linguistic generalization rather than raw throughput. Sparse expert activation, attention pruning, and shared parameterization mitigate excessive resource use, ensuring that the framework remains scalable for medium-to-large corpora. For deployment in resource-constrained environments, further optimization—such as expert pruning, graph sampling, or contrastive-distillation techniques—can reduce inference cost while retaining accuracy. These considerations establish TEGAA as a computationally balanced model that trades marginal increases in training time for significant improvements in task-specific performance and generalization capability (Table 9).

**Table 9:** Computational efficiency comparison.

Model	Training FLOPs (TFLOPs/epoch)	Training memory (GB)	Training time (hours)	Inference FLOPs (GFLOPs/sample)	Inference latency (ms/sample)	F1-score (%)
TEGAA	1.2–1.5	6.5–7.2	12–15	0.9–1.1	45–60	88.5
IANN (Liu and Shen, 2023)	0.8–1.0	3.2–4.0	4–6	0.4–0.5	20–30	85.1
ASHGAT (Ouyang et al., 2024)	0.9–1.1	4.5–5.0	6–8	0.6–0.7	30–40	81.0
BERT unified framework (Murtadha et al., 2024)	1.0–1.2	5.0–5.5	8–10	0.7–0.8	25–35	84.2
Enhanced KNN (Benarafa et al., 2023)	0.6–0.8	2.5–3.0	3–4	0.2–0.3	15–20	78.6
MSRL-Net (Hu et al., 2023)	0.7–0.9	3.5–4.2	5–6	0.3–0.4	18–25	82.3

### 5.8 Broader impact

Beyond implicit aspect detection in text analysis, the broader impact of TEGAA lies in enhancing the model’s ability to uncover underlying sentiments and aspects in text data. TEGAA significantly improves the interpretability and understanding of large-scale unstructured text, such as customer reviews, social media posts, and forum discussions. This deeper insight benefits industries like customer service, marketing, and social listening, enabling businesses and organizations to gain precise knowledge about user preferences, key focus areas, and emerging trends. Moreover, the integration of dynamic expert transformers and graph-based attention mechanisms holds potential to influence other NLP domains, including machine translation, question-answering, and summarization, by providing a more nuanced grasp of context and relationships within textual data. On a societal level, the ability to detect implicit dimensions in online discussions can foster a more detailed understanding of public opinion, empowering policymakers, researchers, and companies to respond effectively to the needs and concerns of diverse communities.

The TEGAA model is designed to handle noisy inputs through several architectural mechanisms. Semantic Contrastive Learning (SCL) helps separate meaningful contextual patterns from spurious or corrupted signals, enhancing the discriminability of embeddings even in the presence of noise. The Dynamic Adaptive Expert Engine (DAEE) further strengthens robustness by dynamically routing ambiguous or degraded embeddings to specialized expert sub-networks, ensuring that task-relevant information is preserved. Additionally, the context-aware graph attention mechanism in GE-HAD selectively emphasizes contextually important nodes, mitigating the impact of irrelevant or noisy tokens. While empirical validation on controlled-noise datasets is planned as a future extension—using synthetic perturbations such as lexical masking, random token insertion, and sentiment-flip modifications—these design features collectively provide intrinsic resilience to noisy and imperfect data, improving the reliability of implicit aspect detection in real-world applications.

## 6 Conclusion

Implicit aspect detection is inherently challenging due to aspect ambiguity, data imbalance, contextual noise, and aspect drift in unstructured text. The TEGAA framework addresses these issues through a principled integration of Dynamic Expert Transformers (DET) with a Dynamic Adaptive Expert Engine (DAEE), Semantic Contrastive Learning (SCL), and a Graph-Enhanced Hierarchical Aspect Detector (GE-HAD). DET dynamically routes token embeddings to specialized expert sub-networks, reducing the impact of noisy or irrelevant patterns while capturing complex syntactic and semantic dependencies. SCL enhances feature discriminability by aligning semantically related samples and separating unrelated ones, effectively mitigating the sparsity of implicit cues. GE-HAD constructs multi-level hierarchical graphs with context-aware graph attention, Attention Sinks, and Pyramid Pooling, capturing both local and global dependencies to resolve ambiguity and maintain stable predictions across varying contexts. The iterative feedback loop between DET and GE-HAD allows continuous refinement of embeddings and attention weights, improving adaptability to aspect drift. Extensive evaluation on Mobile Reviews, SemEval14, and Sentihood datasets demonstrates that TEGAA consistently outperforms state-of-the-art transformer- and graph-based models, achieving F1-scores above 0.88, precision above 0.89, recall above 0.87, accuracy exceeding 89%, and AUC values above 0.89, validating its robustness, scalability, and practical utility. Future work will focus on further improving computational efficiency for large-scale and real-time deployment, developing adaptive attention mechanisms that dynamically adjust to text complexity, integrating domain-specific knowledge for specialized applications such as healthcare, legal, and finance, extending the model to multilingual and cross-lingual scenarios, and exploring active learning or iterative feedback strategies to continuously refine performance on evolving data streams. These advancements will enhance TEGAA’s generalization, interpretability, and applicability across diverse NLP tasks and real-world environments.

## Data Availability

The datasets presented in this study can be found in online repositories. The names of the repository/repositories and accession number(s) can be found at: https://ieee-dataport.org/documents/mobile-review-dataset-aspect-level-sentiment-analysis.
